# Antibacterial plant combinations prevent postweaning diarrhea in organically raised piglets challenged with enterotoxigenic *Escherichia coli* F18

**DOI:** 10.3389/fvets.2023.1095160

**Published:** 2023-04-03

**Authors:** Kevin Jerez-Bogota, Martin Jensen, Ole Højberg, Paul Cormican, Peadar G. Lawlor, Gillian E. Gardiner, Nuria Canibe

**Affiliations:** ^1^Department of Food Science, Aarhus University, Aarhus, Denmark; ^2^Department of Animal and Veterinary Sciences, Aarhus University, Tjele, Denmark; ^3^Animal Bioscience Research Centre, Teagasc Grange, Meath, Ireland; ^4^Pig Development Department, Teagasc Animal and Grassland Research and Innovation Centre, Fermoy, Ireland; ^5^Department of Science, Eco-Innovation Research Centre, Southeast Technological University, Waterford, Ireland

**Keywords:** antibacterial plant, apple pomace, blackcurrant, diarrhea, *Escherichia coli*, garlic, microbiota, organic pigs

## Abstract

Antibiotics and zinc oxide restrictions encourage the search for alternatives to combat intestinal pathogens, including enterotoxigenic *Escherichia coli* (ETEC), a major cause of postweaning diarrhea (PWD) in pigs. PWD causes important economic losses for conventional and organic farming. This study investigated the effect of dietary supplementation with garlic and apple pomace or blackcurrant on infection indicators and the fecal microbiota of organic-raised piglets challenged with ETEC-F18. For 21 days, 32 piglets (7-weeks-old) were randomly assigned to one of four groups: non-challenge (NC); ETEC-challenged (PC); ETEC-challenged receiving garlic and apple pomace (3 + 3%; GA); ETEC-challenged receiving garlic and blackcurrant (3 + 3%; GB). ETEC-F18 was administered (8 mL; 10^9^ CFU/ml) on days 1 and 2 postweaning. The 1st week, PC had lower average daily gain than those in the NC, GA, and GB groups (*P* < 0.05). NC pigs showed neither ETEC-F18 shedding nor signs of diarrhea. The PC group had higher diarrhea incidence and lower fecal dry matter than NC (≈5–10 days; 95% sEBCI). The GA and GB groups showed reduced ETEC-F18 and *fedA* gene shedding, higher fecal dry matter, and lower diarrhea incidence than the PC (≈5–9 days; 95% sEBCI). The NC, GA, and GB had normal hematology values during most of the study, whereas the PC had increased (*P* < 0.05) red blood cells, hemoglobin, and hematocrit on day 7. Haptoglobin and pig-MAP increased in all groups, peaking on day 7, but PC showed the greatest increase (*P* < 0.05). The fecal microbiota of PC pigs had reduced α-diversity (day 7; *P* < 0.05) and higher volatility (days 3–14; *P* < 0.05). *Escherichia, Campylobacter*, and *Erysipelothrix* were more abundant in the PC than in the NC, GB, and GA groups (log_2_FC > 2; *P* < 0.05), whereas *Catenibacterium, Dialister*, and *Mitsoukella* were more abundant in the NC, GB, and GA than in the PC group (log_2_FC > 2; *P* < 0.05). *Prevotella* and *Lactobacillus* were more abundant in the GB group (log_2_FC > 2, *P* < 0.05). In conclusion, dietary supplementation of GA and GB limited ETEC proliferation, reduced PWD, and beneficially impacted the fecal microbiota's diversity, composition, and stability.

## 1. Introduction

Enterotoxigenic *Escherichia coli* (ETEC) expressing F4 and F18 fimbriae is recognized as the main causative agents of postweaning diarrhea (PWD). It is a substantial cause of morbidity and mortality in young pigs, resulting in economic loss due to the cost of treatment, impaired growth, and animal loss (1, 2).

Antibiotics and medical levels of zinc oxide have long been used to treat and prevent PWD in conventionally produced pigs. However, antimicrobial resistance and environmental issues now limit their use (3). Since 2006, the European Union has banned all antibiotic growth promoters and has recently adopted new regulations limiting the amount of zinc in animal feed to 150 ppm and restricting any form of routine antibiotic use (4). Alternative strategies and/or additives are therefore needed to sustainably reduce the need for antibiotic use while promoting pig health, growth, and welfare.

The prevalence of PWD is also a challenge in organic farming systems, where the use of antibiotics and other synthetic drugs is restricted (5). Based on prescription data for Danish organic pig production in 2016, Kruse et al. (6) reported that 65% of antimicrobial treatment doses in weaners were prescribed for gastrointestinal diseases, accounting for the majority of antibiotic usage on these farms. High pH and reduced stomach emptying in piglets after weaning have been linked to pathogen proliferation and PWD (7). The proliferation and attachment of ETEC to the pig intestinal mucosa is a crucial step in the pathogenesis of PWD (1). Hence, dietary strategies that selectively diminish ETEC growth or limit adhesion to enterocytes, or potentially both, are crucial for reducing PWD (7).

Medicinal plants and related products have been suggested as prophylactic and therapeutic alternatives against gastrointestinal diseases in pigs (8). Indeed, previous research from our group indicated that supplementing piglet diets with *Allium ursinum* (wild garlic) bulbs and *Vaccinium vitis*-*idaea* (lingonberries) elicits antibacterial effects against coliforms along the gastrointestinal tract, while having no effect on lactic acid bacteria counts (9). The bactericidal effect was attributed to the allicin in wild garlic bulbs and the pH-lowering effect of the berries; additionally, *in vitro* screening revealed that these effects were synergistic (9). However, because the supply of wild garlic bulbs and lingonberries is limited (harvested from nature and thus expensive plant resources), it is relevant to determine whether similar effects can be obtained with a combination of more readily available and less expensive plant material with comparable properties.

Garlic (*Allium sativum*) is the most studied plant of the *Allium* species in terms of organosulfur compound concentrations (10). Allicin is formed from garlic and garlic powders when the enzyme, alliinase, is activated by crushing cloves or wetting powder, allowing alliin to be converted to allicin and other allyl thiosulfinates (11). Apple pomace, a byproduct of mashing and pressing apples (*Malus domestica*) for juice, cider, or puree, has been identified as a valuable source of polyphenols, dietary fiber, and organic acids (12, 13). Apple pomace has been reported to have a pH ≈ 4 (12, 14), varying by cultivar and maturity of apples at harvest. Blackcurrant (*Ribes nigrum*) is a perennial shrub native to central Europe and northern Asia (15) that is cultivated in gardens and berry orchards for juice production and has been shown to inhibit *E. coli* (16). Blackcurrants contain high levels of pectic polysaccharides, anthocyanins, and organic acids. The pH of blackcurrants is ≈ 2.7 (17), but this varies depending on the cultivar.

As observed by Canibe et al. (9) with the mixture of wild garlic and lingonberries, the combined antibacterial effect of allicin from garlic and the acidifying properties of apple pomace or blackcurrant, are expected to inhibit ETEC proliferation in the gut of weaned piglets. We hypothesized that supplementing piglets with garlic and apple pomace, or garlic and blackcurrant alleviates clinical signs of PWD by selectively targeting ETEC and promoting a healthy gut microbiota. The objective of this study was to assess infection indicators and fecal microbiota dynamics in organically reared weaned piglets following a postweaning ETEC F18 challenge and dietary supplementation with garlic powder in combination with apple pomace or blackcurrant powder.

## 2. Materials and methods

The study was performed at the Department of Animal and Veterinary Sciences of Aarhus University (Denmark). The animal and experimental procedures were approved by the Danish Animal Experiments Inspectorate, Ministry of Food, Agriculture and Fisheries, Danish Veterinary and Food Administration (License 2017-15-0201-01270). Animal care and housing were in accordance with Danish laws and regulations governing the humane care and use of animals in research.

### 2.1. Antibacterial plant preparation

Fresh organically cultivated garlic (Therador cultivar) was obtained from Årslev Research Center (Department of Food Science, Aarhus University, Denmark), and a small amount from imported from GIE l'Ail Drômois (Eurre, France). The garlic cloves were cut into chips using a cutter (AK-RAMON; Talleres Ramon, S.L., Barcelona, Spain) and dried in shallow layers at 40°C in a ventilated oven (Binder GmbH, Tuttlingen, Germany) before being ground into a fine powder (Ø = 0–1 mm) in a centrifugal mill (ZM200; Retsch GmbH, Haan, Germany). The garlic powder was stored in sealed plastic bags at −20°C until it was used. This preparation of the dry powder guaranteed the presence of alliin and alliinase, which upon wetting results in enzymatic allicin production (i.e., allicin is generated when pigs ingest the powder). To confirm activity, the allicin produced by the garlic powder upon wetting was determined. Samples were analyzed using high-pressure liquid chromatography (HPLC). Briefly, a 50 mg sample was incubated in water (5 ml, MilliQ) at room temperature for 10 min to allow the alliinase enzyme to convert alliin to allicin. Following sedimentation, 1 ml supernatant from each sample was mixed with 1 ml methanol (HPLC grade) and then centrifuged for 3 min at 10,621 g and filtered through a 0.4 μm nylon filter into HPLC vials. A standard curve (*R*^2^ = 0.995) to quantify the potential allicin content expressed as mg alliin Eq/g dry powder was created by spiking different known amounts of alliin (S-Allyl-L-cysteine sulfoxide, 74264; SigmaAldrich, Søborg, Denmark) against a standard garlic sample. This method of quantification (18) was used since reference compounds of pure allicin are not available due to low stability. Measurement was performed on a Nexera X2 LC30AD HPLC system (Shimadzu, Kyoto, Japan) equipped with an SPDM20A prominence diode array detector and a Hypersil GOLD column (4.6 × 250 mm; 5 μm; Thermo Fisher Scientific, Waltham, Massachusetts, USA).

Apple pomace from juice pressing was obtained from Orskov Foods (Ørbæk, Denmark). The apples were mainly of the cultivar Elstar and cultivated organically in their own orchard. The apple pomace was immediately frozen after pressing and stored at −20°C until use. The frozen apple pomace was thawed and ground in a wet grinder (GM300, Retsch GmbH) to ensure that the seeds were macerated, allowing for possible enzymatic cyanide formation, before drying to evaporate any accumulated cyanide. Drying was done on shelves in a Memmert drying cabinet at 60°C during 5 days. Dry apple mash was milled into a fine powder (Ø = 0–1 mm) using a centrifugal mill (ZM200, Retsch GmbH). The powder was stored in sealed plastic bags at −20°C until use. Freeze dried blackcurrant (unspecified cultivar) powder (Ø = 0–1 mm) was derived from organic berries grown in Lithuania, and provided by the company Berrifine (Ringsted, Denmark). The pH of the apple and blackcurrant powders was tested in a 25% (w/v) Milli-Q water solution.

Various concentrations of the powders were tested alone or in combination for their ability to kill or inhibit the growth of ETEC in porcine gastric digesta (9). Based on the expected concentration of the plant combinations in the stomach upon consumption, the results from the *in vitro* testing were used to determine the appropriate inclusion level of garlic, apple pomace, and blackcurrant powders in the feed (data not shown).

### 2.2. Animals, experimental design, and diets

Thirty-two piglets (7-weeks-old; body weight = 20.14 ± 1.5 kg) born from four organically raised sows (TN70 Topigs; Norsvin, Esbjerg Ø, Danmark) were selected for the study (eight piglets per sow, mixed sex). The sows were confirmed to be homozygote carriers of the dominant gene (*FUT*1^GG^) encoding ETEC F18 fimbriae receptors by VHL genetics (Wageningen, The Netherlands); thus, piglets were genetically susceptible to ETEC F18 (19). The pigs were obtained at weaning and the study lasted 21 days. The weaned piglets were housed in pens of two littermates. There was no physical contact between pigs housed in different pens. Treatment groups were housed in different rooms (similar in design) to avoid cross contamination. The rooms were climate-controlled (20 ± 0.4°C).

Two piglets, housed in the same pen, from each sow were randomly assigned to one of four treatments: non-challenged control fed a standard diet (Negative Control, NC); ETEC-challenged control fed a standard diet (Positive Control, PC); ETEC-challenged fed the standard diet supplemented with garlic and apple pomace (3 + 3% w/w, GA); and ETEC-challenged fed the standard diet supplemented with garlic and blackcurrant (3 + 3% w/w, GB). Treatments were balanced by initial body weight.

The diets followed the guidelines for organic pig production and were formulated to meet the Danish nutrient requirement standards of pigs (20). The ingredient composition is presented in Supplementary Table 1.

### 2.3. Challenge with enterotoxigenic *Escherichia coli* and sampling procedures

The ETEC strain (O138 F18-ETEC 9910297-2STM) expressing F18 fimbriae, heat-stable enterotoxin b (STb); heat-labile enterotoxin (LT); enteroaggregative *E. coli* heat-stable enterotoxin 1 (EAST1), and Shiga toxin type 2e (Stx2e), was isolated from the intestinal content of a pig with PWD and was provided by the Danish Veterinary Institute (Copenhagen, Denmark). The ETEC F18 was found to be hemolytic (on blood agar). Enterotoxigenic *E. coli* F18 colonies from blood agar plates were transferred to BHI broth and incubated at 37°C for 5 h with constant shaking (150 rpm). Following centrifugation (12,000 rpm, 10 min), the bacteria were diluted in 0.9% NaCl and the bacterial load was adjusted to OD_600_ = 1.0, corresponding to 10^9^ CFU/ml. The concentrations of the bacterial suspension were confirmed by plate count (blood agar). On days 1 and 2 after weaning, the PC, GA, and GB pigs were orally challenged with 7–8 ml of 0.9% NaCl solution containing ≈10^9^ CFU/ml of ETEC F18. The NC pigs were given 7–8 mL of 0.9% NaCl orally. To administer the solutions, a polyethylene tube was connected to a syringe and placed in the mouth of the piglet.

The pigs were monitored daily for signs of illness, such as diarrhea, lethargy, and dehydration. Individual body weight was recorded at weaning, and on days 7, 14, and 21 postweaning to estimate average daily gain (ADG) and daily feed disappearance per pen was recorded for calculation of average daily feed intake (ADFI). Feed efficiency was then estimated as weight gain to feed intake ratio (G:F).

Fecal samples were collected on the weaning day, daily for the 1st week, and three times a week for the last 2 weeks of the study. The fecal samples were scored on a 7-point scale for consistency (1: hard, dry and cloddy; 2: firm; 3: soft but able to retain some shape; 4: soft and unable to retain any shape; 5: watery and dark; 6: watery and yellow; 7: foamy and yellow) with scores 4 to 7 defined as clinical signs of diarrhea (21). Samples were kept on ice until they were divided into three aliquots for genomic analyses (−80°C), dry matter (−20°C), and ETEC F18 enumeration (conducted immediately).

Individual blood samples were collected from the jugular vein at weaning, and days 3, 5, 7, 14, and 21 postweaning (1, 3, 5, 12, and 19 days after second infection) in EDTA-containing collection tubes for hematology analysis, and heparinized collection tubes for determination of pig C-reactive protein (CRP), haptoglobin, and pig major acute-phase protein (pigMAP) concentrations. Hematology parameters were analyzed immediately after collection. Plasma was obtained from heparinized tubes that were stored on ice and centrifuged (2,000 × *g*, 10 min) within 2 h following collection. Plasma was stored at −20°C until acute phase proteins were analyzed.

### 2.4. Analytical methods

The acid-binding capacity at pH 4 (ABC-4) of the diets was determined as described by Lawlor et al. (22). Dry matter (DM) of diets was determined by drying to constant weight at 103°C (≈20 h), ash according to method 923.03 AOAC (23). Gross energy was measured on a 6300 Automatic Isoperibol Calorimeter system (Parr Instruments, Moline, IL, USA). The crude fat was determined by HCl-Bligh and Dyer extraction as described by Jensen (24). Crude protein (N × 6.25) was determined by the Dumas method (25). Amino acids in diets were determined using the European Economic Community methods (26). The dietary content of sugars (glucose, fructose and sucrose), fructans, starch, soluble and insoluble non-starch polysaccharides, and Klason lignin were measured as described in Knudsen (27).

The dry matter content of the feces (F-DM) was determined by freeze-drying the samples to a constant weight. To determine ETEC counts, ~1–3 g of feces were suspended in a peptone solution (1:10, w/v) and homogenized with a bag blender (Smasher; Biomérieux, Marcy-l'Étoile, France). Serial 10-fold dilutions were prepared and 100-μL aliquots were spread-plated on blood agar (Columbia blood agar with sheep blood medium, Thermo Fisher Scientific, Waltham, Massachusetts, USA) and incubated aerobically overnight at 37°C. Hemolytic bacteria were counted using a manual colony counter and the count expressed as CFU/g feces. Blood agar plates with hemolytic colonies were stored at 5°C until ETEC F18 serotyping was performed using the slide agglutination test with type-specific antisera (SSI Diagnostica A/S, Hillerød, Denmark) on five colonies per plate. The limits of detection were 10^4^ CFU/g on days 1 and 11–21, and 10^5^ CFU/g feces on days 2–9; the higher detection limit was applied on days where the counts were likely to increase.

Quantitative polymerase chain reaction (qPCR) was used for quantification of the gene encoding the F18 fimbriae (*fedA* gene) and STb toxin (*estB* gene) in fecal samples. Briefly, DNA was extracted from fecal samples using the NucleoSpin 96 DNA Stool kit (Macherey-Nagel, Düren, Germany). The concentration of genomic dsDNA was measured using the Qubit Broad Range Assay Kit (Thermo Fisher Scientific, Waltham, Massachusetts, USA) on an Invitrogen Qubit 4.0 Fluorometer (Thermo Fisher Scientific, Waltham, Massachusetts, USA). Following extraction, qPCR was performed on a ViiA 7 real-time PCR system (Applied Biosystems, Waltham, Massachusetts, USA) using a MicroAmp Optical 384 well reaction plate (Applied Biosystems, Waltham, Massachusetts, USA). The qPCR reactions contained 5 μL of Maxima SYBR Green/ROX qPCR Master Mix (Thermo Fisher Scientific, Waltham, Massachusetts, USA), the F18 and STb primers at a concentration of 0.3 mM, 2 μL of template DNA and water to a final volume of 10 μL. All samples were analyzed in triplicate and the data generated in QuantStudio real-time PCR Software v1·4. Primer sequences and qPCR specific settings are presented in Supplementary Table 2.

Complete blood counts were determined using a ProCyte Dx Hematology analyzer (IDEXX B.V., Hoofddorp, Netherlands). Blood plasma haptoglobin was determined using the PHASE Haptoglobin Assay Kit (TP801; Tridelta Developments Ltd., Kildare, Ireland). The CRP and Pig-MAP were determined by particle enhanced immune turbidimetry, using the Turbovet pig CRP and Turbovet Pig-MAP kits (Acuvet Biotech, Zaragoza, Spain). The determinations were conducted using an ADVIA 1800 Clinical Chemistry System autoanalyzer (Siemens Healthineers AG, Erlangen, Germany).

### 2.5. Fecal microbiota profiling

Fecal samples from days 1, 3, 7, 14, and 21 postweaning were used for microbiota profiling. Total microbial DNA was extracted from the samples as previously stated for qPCR analyses and used for bacterial profiling using 16S rRNA gene amplification. Only samples with a DNA concentration >7 ng/μl were used for PCR amplification and for paired-end sequencing of the 16S rRNA V3-V4 region (250-bp paired-end raw reads) on the Illumina platform NovaSeq 6000 (Illumina, San Diego, CA, United States). Library preparation, DNA quality control (Agarose Gel Electrophoresis; 5400 Fragment Analyzer, Agilent, USA) and sequencing was performed by Novogene (Novogene UK Company Limited, Cambridge, United Kingdom).

The raw amplicon sequencing data was processed into the table of exact amplicon sequence variants (ASV) using the DADA2 pipeline (28) in R (version 4.2) for the identification of ASVs. Reads were filtered and trimmed based on quality, de-noised, and merged, then chimeras removed, and taxonomy was assigned to each ASV using a naive Bayesian classifier method against the SILVA reference database v138 (29). Subsequent filters included the removal of read lengths shorter than 398, non-bacterial and cyanobacteria reads, and ASVs with prevalence <0.005 across all samples.

Analyses were performed after rarefying at 90% of minimum sampling depth, except for α-diversity measures, where unrarefied reads were used. The α-diversity (observed, Shannon, and inverse Simpson) and β-diversity measures (weighted UniFrac dissimilarity matrices) were calculated using phyloseq (30) in R (version 4.2.1). To assess community structure changes over time, microbiota volatility (31) was measured as the weighted UniFrac distance between the individual community structure from the previous individual timepoint. Differential abundance analysis was conducted using the DEseq2 package (32) in R (version 4.2.1) and involved a threshold of 0.1% relative abundance and 10% prevalence.

### 2.6. Statistical analyses

Differences in pig growth performance were assessed using ANOVA. The model included treatment as a fixed effect, initial body weight was included as a covariate for ADG and ADFI, and pen and sow effects were included as random effects (PROC MIXED, SAS Studio). Assumptions of normality and homogeneity of variance were confirmed. *P*-values were adjusted for multiple comparisons using the Holm-Bonferroni adjustment.

The remaining statistical analyses were carried out in R (version 4.2.1). Unless otherwise specified, *P-*values were adjusted for multiple comparisons using the Benjamini-Hochberg method and differences were considered significant if *P* < 0.05.

Hierarchical generalized additive models (33) were used to analyze F-DM, fecal scores, ETEC counts, and qPCR data using the mgcv package (34). Fecal scores were modeled using ordered categorial distribution. Model diagnostics were assessed *via* the appraise function of the gratia package (35). The models included treatment as parametric term, independent smooths over time for each treatment, interaction smooths over time for each pig, and random effects of pen and sow. Pairwise differences between treatment groups against the PC group were assessed *via* 95% simultaneous empirical Bayesian confidence intervals (sEBCI) as described by Mundo et al. (36).

Results of acute phase proteins, and microbiota α-diversity measures and volatility were analyzed using generalized linear mixed models with day, treatment and their interaction as fixed effects, and random slopes by subjects, pen and sow were also included as random effects. The models were fit using the glmmTMB package, adequate distribution and covariance structure were selected by best fit and regression diagnostics using the performance package (37).

Microbiota β-diversity was examined by non-metric multidimensional scaling (NMDS) ordination and compared using permutational multivariate analysis of variance (PERMANOVA) in the vegan package (38). Differentially abundant taxa between treatments were identified using a time course experiment strategy in the DeSeq2 workflow (32). Briefly, a likelihood ratio test with a model including day, treatment, and their interaction was performed. Wald test was then used to evaluate taxa in pairwise comparisons against the PC group within day. The threshold for classification as differentially abundant taxa was log_2_ fold-change (log_2_FC) > 2 and adjusted *P* < 0.05. The differentially abundant taxa were clustered using the pheatmap package.

## 3. Results

The potential allicin content in the garlic powder was found to be 44.1 ± 6.0 mg alliin Eq/g dry powder (*n* = 3). The pH of the apple pomace powder was 3.28 ± 0.01 and the pH of the blackcurrant powder was 2.81 ± 0.01. The initial pH (before ABC-4 measurement) of the Control (NC and PC), GA and GB diets were 6.39 ± 0.01, 6.16 ± 0.02, and 6.03 ± 0.01, respectively (n = 3). The ABC-4 of the Control (NC and PC), GA, and GB diets were 454.1, 388.8, and 323.2 mEq, respectively. The chemical composition of the GA and GB experimental diets was similar to that of the diet fed to NC and PC pigs (Table 1). The GA and GB diets had a greater content of fructans than the control diet i.e., 1.61 and 1.45 vs. 0.04%, respectively.

**Table 1:** Analyzed composition of experimental diets (dry matter basis).

**Item**	**Diets^a^**		
	**NC—PC**	**GA**	**GB**
Moisture, %	10.64	10.12	9.57
Crude protein, %	21.40	21.77	21.21
Gross energy, kcal/kg	4,476	4,456	4,451
Crude fat, %	4.06	4.15	3.79
Ash, %	4.96	5.07	5.20
**Indispensable amino acids, %**			
Arginine	1.25	1.23	1.21
Histidine	0.48	0.48	0.46
Isoleucine	0.92	0.92	0.88
Leucine	1.59	1.57	1.52
Lysine	1.36	1.35	1.33
Methionine	0.40	0.38	0.38
Phenylalanine	1.02	1.00	0.97
Threonine	0.83	0.83	0.80
Valine	1.13	1.12	1.08
**Dispensable amino acids, %**			
Alanine	0.98	0.97	0.94
Aspartic acid	1.86	1.87	1.78
Cysteine	0.36	0.34	0.34
Glutamic acid	3.70	3.64	3.59
Glycine	1.00	0.98	0.96
Proline	1.29	1.28	1.25
Serine	1.02	1.00	0.98
**Carbohydrates, %**			
Fructose	0.11	0.81	0.78
Glucose	0.25	0.77	0.53
Sucrose	2.74	3.12	3.02
Fructans	0.04	1.61	1.45
Starch	43.44	44.23	44.16
*S*−*NSP*^b^	3.57	2.35	3.60
Rhamnose	0.02	0.03	0.04
Fucose	0.01	0.01	0.02
Arabinose	0.59	0.51	0.67
Xylose	0.90	0.16	0.51
Mannose	0.20	0.18	0.22
Galactose	0.29	0.31	0.35
Glucose	1.27	0.65	1.36
Uronic acids	0.28	0.49	0.43
*I*−*NSP*^c^	11.73	11.88	12.13
Rhamnose	0.02	0.02	0.02
Fucose	0.02	0.02	0.03
Arabinose	1.73	1.63	1.61
Xylose	3.91	3.95	3.86
Mannose	0.26	0.38	0.30
Galactose	0.32	0.33	0.37
Glucose	1.23	1.83	1.31
Uronic acids	0.46	0.49	0.47
Cellulose	3.77	3.25	4.16
Total NSP^d^	15.29	14.23	15.73
Klason lignin	3.00	3.41	2.79
Dietary fiber^e^	18.33	19.25	19.96

a NC, non-challenge, standard diet; PC, challenged, standard diet; GA, challenged, garlic and apple pomace supplementation (3 + 3%); GB, challenged, garlic and blackcurrant supplementation (3 + 3%).

b Soluble non-starch polysaccharides.

c Insoluble non-starch polysaccharides.

d Total non-starch polysaccharides (S-NSP + I-NSP).

e Total non-starch polysaccharides + lignin + fructans.

All pigs started the study in good health. The NC pigs were in good health throughout the study, whereas one pig from the PC group died 5 days postweaning and two pigs from the GA group in the same pen died on days 6 and 7, respectively. The deceased pigs had high counts of ETEC in the feces (>10^8^ CFU/g feces; >10^5^ *fedA* gene copies/g feces) and displayed signs of diarrhea (Fecal score = 6; <10% F-DM).

### 3.1. Growth performance

There was no overall effect (*P* > 0.05) of treatment on body weight, ADG, ADFI and G:F during the experiment (Table 2). During the 1st week after weaning, the PC pigs had lower ADG than NC pigs, but no difference in ADFI was detected between treatments, resulting in PC having the lowest G:F of all treatments. When compared to the PC group, the GA and GB pigs had higher ADG in the 1st week. In contrast, the PC group had the highest ADG during the following week. The ADG and ADFI of the GA and GB pigs did not differ from those of the NC pigs during any of the calculated periods. During the 1st week of the study, the GA pigs had the highest G:F, followed by NC and GB and then finally PC.

**Table 2:** Effect of postweaning enterotoxigenic *E. coli* (ETEC) F18 challenge and dietary supplementation with plant combinations on postweaning performance of organic weaners.

**Item**	**Treatment^d^**				**SEM^e^**	***P*-value**
	**NC**	**PC**	**GA**	**GB**		
**Body weight, kg**						
Weaning	20.17	19.98	20.58	19.81	1.451	0.987
Day 7	23.29	22.19	22.79	22.55	1.842	0.976
Day 14	28.32	27.78	27.30	26.92	2.132	0.966
Day 21	34.11	33.82	32.39	31.74	2.797	0.902
**Average daily gain, g/d**						
Day 0–7	441.3^a^	234.8^b^	432.3^a^	395.3^ab^	43.085	0.007
Day 7–14	714.5^ab^	840.1^a^	675.9^b^	628.3^b^	33.933	0.012
Day 14–21	823.5	841.7	781.8	692.6	55.745	0.486
Overall	660.9	633.1	607.4	569.8	48.542	0.333
**Feed intake, g/d**						
Day 0–7	793.1	784.7	660.6	746.2	35.79	0.141
Day 7–14	1,417	1,429.5	1,377.6	1,245.9	87.61	0.303
Day 14–21	1,742	1,757.1	1,560.3	1,589.9	75.45	0.169
Overall	1,318.7	1,281.9	1,200.3	1,156.7	61.44	0.291
**G:F^f^**						
Day 0–7	0.56^b^	0.35^c^	0.72^a^	0.53^b^	0.049	<0.001
Day 7–14	0.51	0.53	0.52	0.51	0.016	0.946
Day 14–21	0.47	0.48	0.5	0.46	0.015	0.809
Overall	0.5	0.48	0.51	0.49	0.017	0.736

abc Within a row, values that do not share a common superscript differ (*P* < 0.05), Holm-Bonferroni adjustment.

d NC, non-challenge (*n* = 8), standard diet; PC, challenged, standard diet (0–7 d, *n* = 8; 7–21 d, *n* = 7); GA, challenged, garlic + apple pomace (3 + 3%; 0–7 d, *n* = 8; 7–21 d, *n* = 6); GB, challenged, garlic + blackcurrant (3 + 3%; *n* = 8).

e Pooled standard error of least squared means.

f Gain to feed ratio (feed efficiency).

### 3.2. Fecal consistency and fecal dry matter

**Figure 1:**
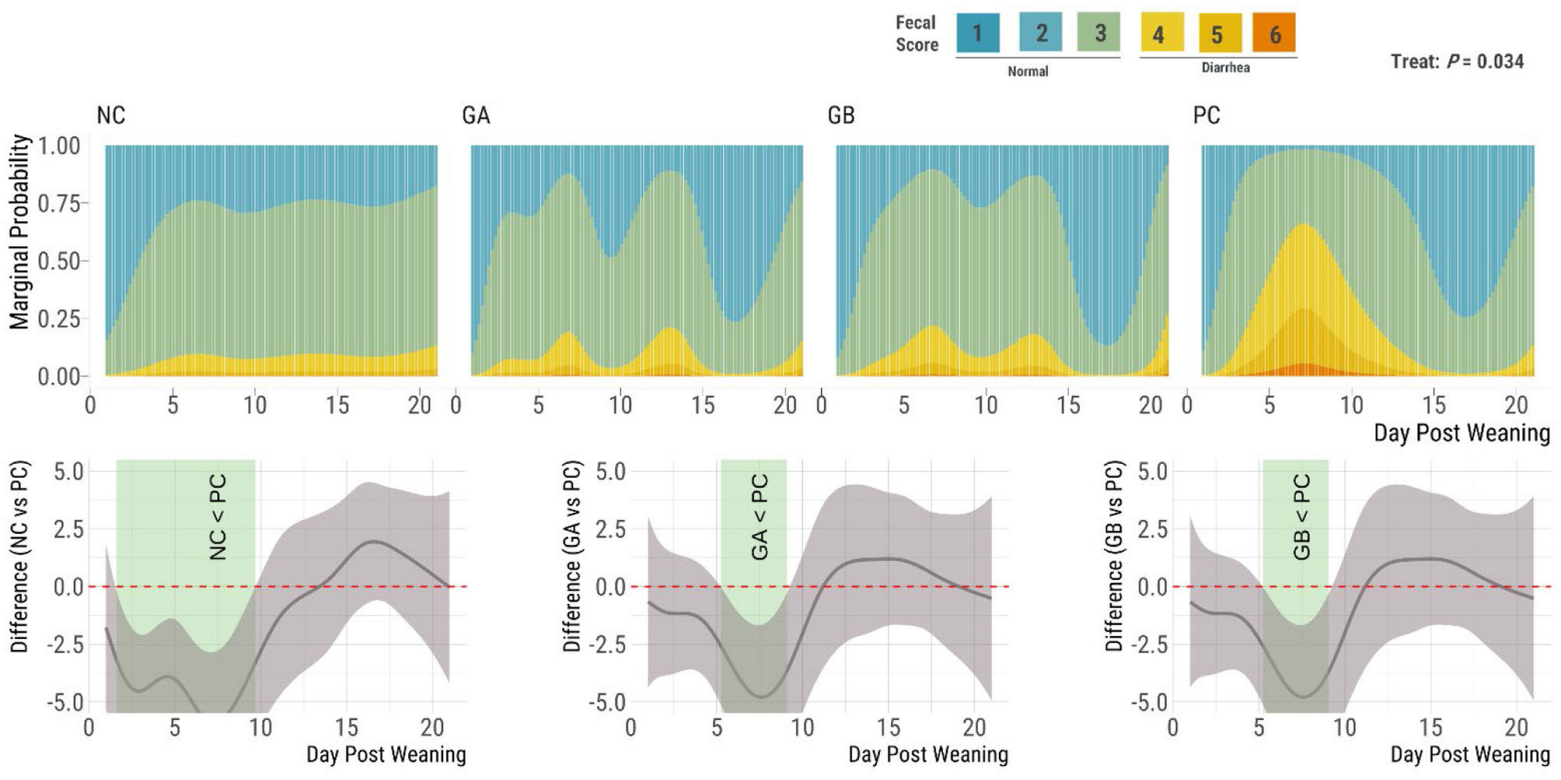
Effect of postweaning enterotoxigenic *E. coli* (ETEC) F18 challenge and dietary supplementation with plant combinations on fecal consistency scores in organic weaner pigs (based on a 7-point scale with scores 4–7 indicating diarrhea). The ETEC F18 was administered orally on days 1 and 2. **(Top panel)** Stacked bar plots for the marginal probability occurrence of each score category over time by treatment. **(Bottom panel)** Pairwise comparisons against PC; significant differences exist where the 95% empirical Bayesian simultaneous confidence interval does not cover 0 (green shade). NC, non-challenge, standard diet (*n* = 8); PC, challenged, standard diet (0–7 d, *n* = 8; 7–21 d, *n* = 7); GA, challenged, garlic + apple pomace (3 + 3%; 0–7 d, *n* = 8; 7–21 d, *n* = 6); GB, challenged, garlic + blackcurrant (3 + 3%; *n* = 8).

The fecal consistency score was influenced by treatment (*P* = 0.034; Figure 1). The cumulative marginal probability of loose stools (scores 4–7) was below 15% overall in the NC pigs, below 20% for the GA and GB groups, and above 50% in the PC group between day ≈5 and 9. The NC group had a higher (95% sEBCI) proportion of firm feces (and fewer soft feces) than the PC group from days 5 to 10, while the GA and GB groups had a higher (95% sEBCI) proportion of firm feces than the PC group from days 6 to 8 postweaning.

**Figure 2:**
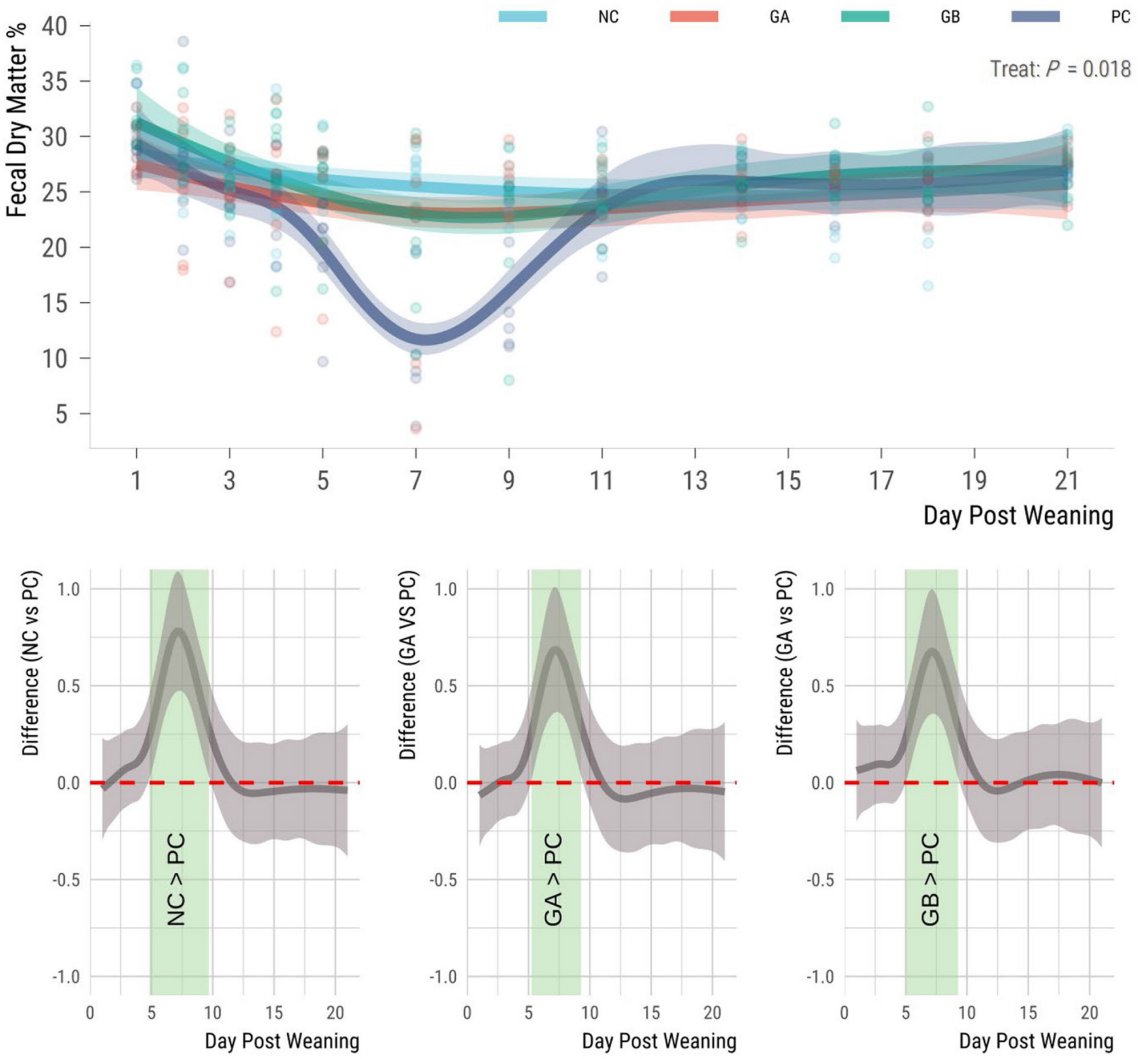
Effect of postweaning enterotoxigenic *E. coli* (ETEC) F18 challenge and dietary supplementation with plant combinations on fecal dry matter (%) of organically reared weaner pigs. **(Top panel)** Lines represent trends for each group; shaded regions are 95% confidence intervals. The ETEC F18 was administered orally on days 1 and 2. **(Bottom panel)** Pairwise comparisons against PC; significant differences exist where the 95% empirical Bayesian simultaneous confidence interval does not cover 0 (green shade). NC, non-challenge, standard diet (*n* = 8); PC, challenged, standard diet (0–7 d, *n* = 8; 7–21 d, *n* = 7); GA, challenged, garlic + apple pomace (3 + 3%; 0–7 d, *n* = 8; 7–21 d, *n* = 6); GB, challenged, garlic + blackcurrant (3 + 3%; *n* = 8).

The F-DM was influenced by treatment (*P* = 0.018; Figure 2). The mean F-DM percentage for the NC, GA, and GB groups remained above 20% throughout the study, while the PC pigs had the lowest percentages of F-DM from 5 to 9 days postweaning, dropping below 20%. The F-DM of the NC pigs was higher (95% sEBCI) than that of the PC groups from days 5 to 10. When compared to the PC group the GA and GB pigs had higher (95% sEBCI) F-DM from days 5 to 9 postweaning.

### 3.3. Fecal shedding of enterotoxigenic *Escherichia coli* F18 and virulence factor genes

**Figure 3:**
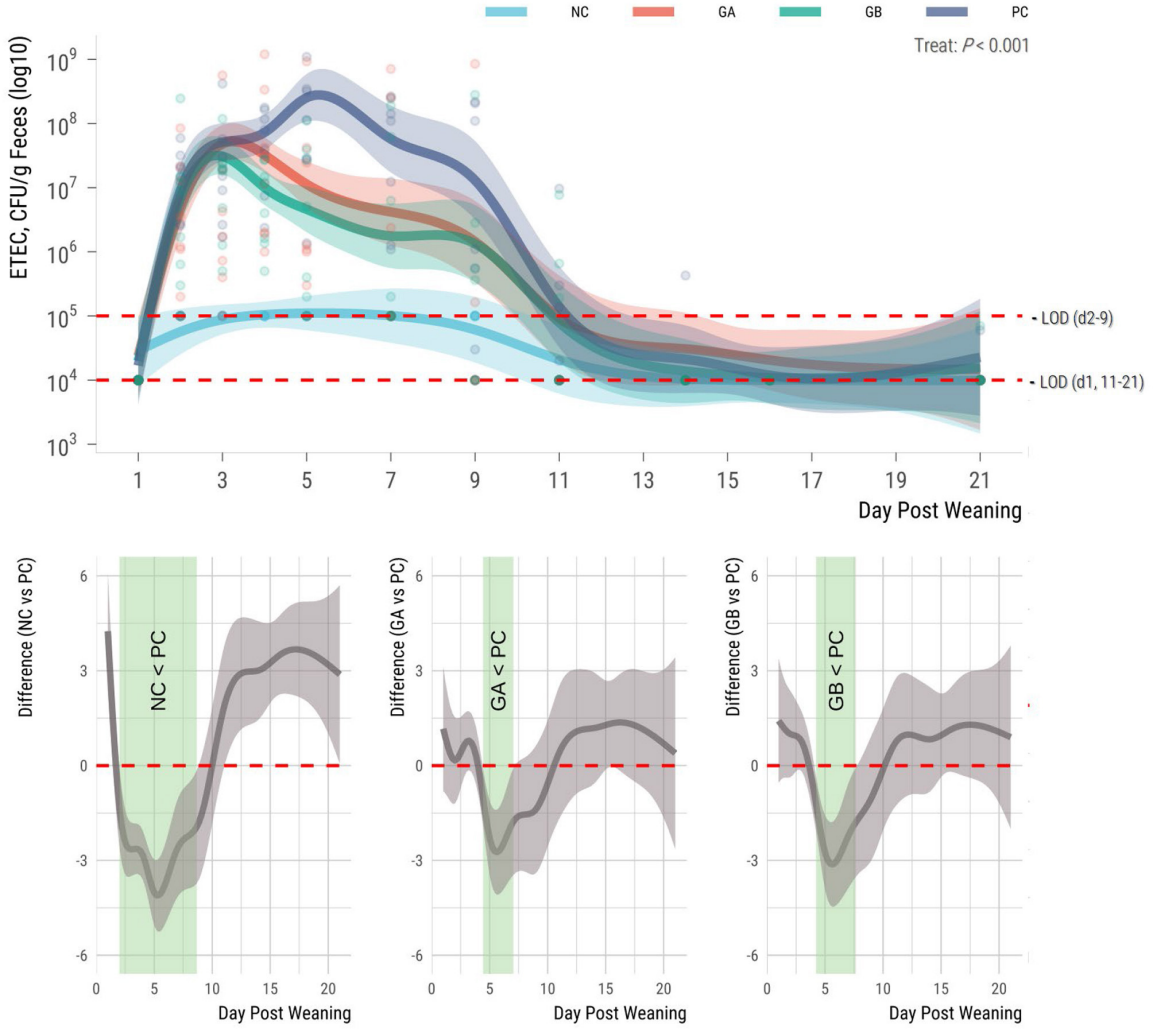
Effect of postweaning enterotoxigenic *E. coli* (ETEC) F18 challenge and dietary supplementation with plant combinations on fecal shedding of ETEC F18 [plate counts; Colony forming units (CFU)] in organically reared weaner pigs. **(Top panel)** Lines represent trends for each group; shaded regions are 95% confidence intervals. The ETEC F18 was administered orally on days 1 and 2. **(Bottom panel)** Pairwise comparisons against PC; significant differences exist where the 95% empirical Bayesian simultaneous confidence interval does not cover 0 (green shade). NC, non-challenge, standard diet (*n* = 8); PC, challenged, standard diet (0–7 d, *n* = 8; 7–21 d, *n* = 7); GA, challenged, garlic + apple pomace (3 + 3%; 0–7 d, *n* = 8; 7–21 d, *n* = 6); GB, challenged, garlic + blackcurrant (3 + 3%; *n* = 8).

The fecal counts of ETEC F18 were influenced by treatment (*P* < 0.001; Figure 3). There was no detectable ETEC F18 in the feces of pigs prior to ETEC challenge. During the study, the ETEC F18 counts in the feces of the NC pigs were around the detection limits; whereas counts in the other groups were detectable from days 2 to 11 and remained stable at low levels thereafter. The ETEC counts in feces of NC pigs were lower (95% sEBCI) than in the PC pigs from days 2 to 9. When compared to the PC group, the GA group had lower counts (95% sEBCI) from days 4 to 7, and the GB group had lower counts from days 4 to 8 postweaning.

**Figure 4:**
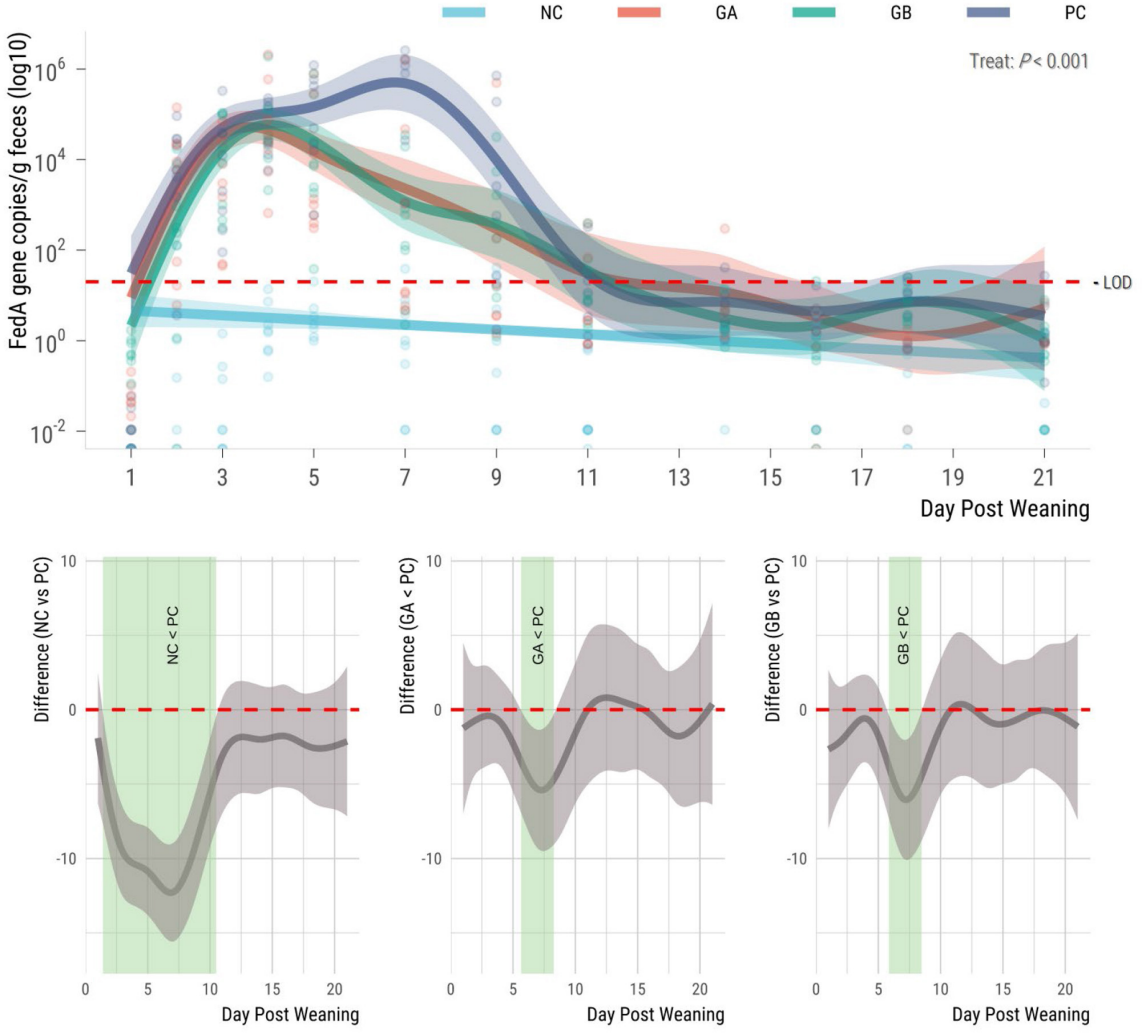
Effect of postweaning enterotoxigenic *E. coli* (ETEC) F18 challenge and dietary supplementation with plant combinations on fecal shedding of the *fedA* gene encoding the ETEC F18 fimbriae type (gene copies/g feces) in organically reared weaner pigs. **(Top panel)** Lines represent trends for each group, shaded regions are 95% confidence intervals. The ETEC F18 was administered orally on days 1 and 2. **(Bottom panel)** Pairwise comparisons against PC; significant differences exist where the 95% empirical Bayesian simultaneous confidence interval does not cover 0 (green shade). NC, non-challenge, standard diet (*n* = 8); PC, challenged, standard diet (0–7 d, *n* = 8; 7–21 d, *n* = 7); GA, challenged, garlic + apple pomace (3 + 3%; 0–7 d, *n* = 8; 7–21 d, *n* = 6); GB, challenged, garlic + blackcurrant (3 + 3%; *n* = 8).

The *fedA* gene was not detectable in the feces of the pigs prior to ETEC challenge, and the copy number was influenced by treatment thereafter (*P* < 0.001; Figure 4). The F18-encoding gene was not present in the NC pigs at any time point during the study. Gene copies increased in all challenged groups from day 2, but the PC group had the highest mean levels of the *fedA* gene. The NC pigs had fewer *fedA* gene copies (95% sEBCI) than the PC pigs from days 2 to 11. When compared to the PC group, the GA and GB groups had fewer (95% sEBCI) gene copies from days 6 to 9 postweaning.

**Figure 5:**
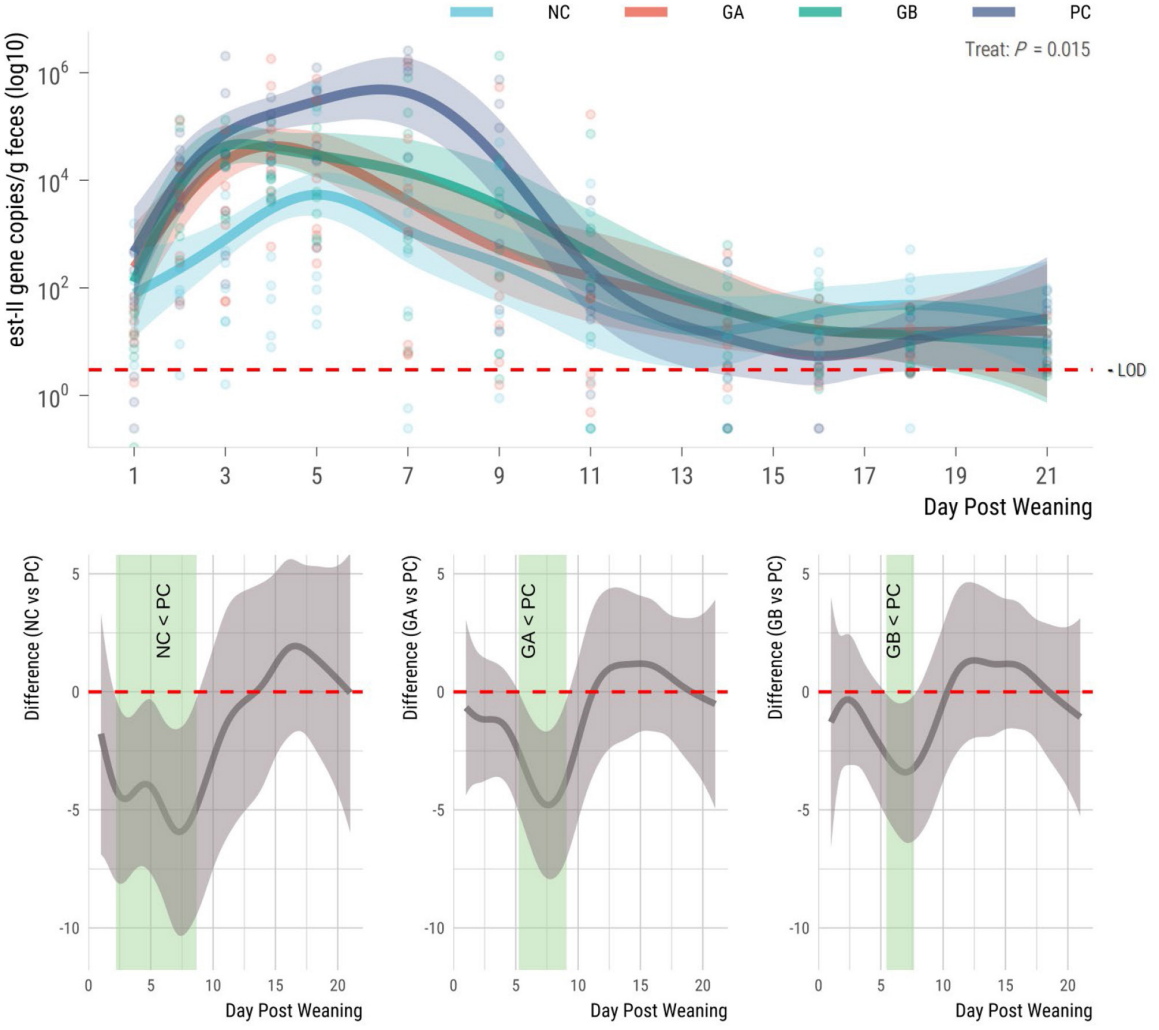
Effect of postweaning enterotoxigenic *E. coli* (ETEC) F18 challenge and dietary supplementation with plant combinations on fecal shedding the *est*-*II* gene encoding the heat-stabile toxin Stb (gene copies/g feces). **(Top panel)** Lines represent trends for each group, shaded regions are 95% confidence intervals The ETEC F18 was administered orally on days 1 and 2. **(Bottom panel)** Pairwise comparisons against PC; significant differences exist where the 95% empirical Bayesian simultaneous confidence interval does not cover 0 (green shade). NC, non-challenge, standard diet (*n* = 8); PC, challenged, standard diet (0–7 d, *n* = 8; 7–21 d, *n* = 7); GA, challenged, garlic + apple pomace (3 +3%; 0–7 d, *n* = 8; 7–21 d, *n* = 6); GB, challenged, garlic + blackcurrant (3 + 3%; *n* = 8).

The *est*-*II* gene (STb toxin) was detectable in all groups prior to ETEC challenge (Figure 5). The shedding of the gene in feces increased in all groups after weaning and differed (95% sEBCI) among groups from days 2 to 7. The NC group had the lowest (95% sEBCI) levels of gene copies during the whole study period. Gene copies increased in all groups beginning on day 2, but the PC group had the highest mean (95% sEBCI) levels of *est*-*II* gene. The NC pigs shed fewer (95% sEBCI) copies of the gene than the PC pigs from days 2 to 9. When compared to the PC group, the GA pigs shed fewer STb-encoding gene copies from days 5 to 9 postweaning (95% sEBCI), and the GB pigs from days 5 to 8 postweaning (95% sEBCI).

### 3.4. Blood hematology

The effect of treatment on the red blood cell count (RBC), hemoglobin (HGB), hematocrit (HCT), mean cell value (MCV), mean cell hemoglobin (MCH), and reticulocyte count was dependent on the day (*P* < 0.001; Table 3). The mean cell hemoglobin content (MCHC) and platelet count were unaffected by the treatments. On the 1st day of the study the GB pigs had lower HCT and reticulocyte count than the NC pigs. By day 3, the reticulocyte count in GA group was less than the NC and PC pigs, and the GB pigs had a reduced reticulocyte count than the NC group. By day 7, the RBC, HBC, and HCT were lower levels in NC, GA, and GB pigs than in PC group. On day 14, HGB levels were reduced in GA and GB pigs, and their reticulocyte count was higher than in NC and PC pigs. Finally, on day 21, the GA and GB pigs had higher reticulocyte counts and reduced RBC, HGB, and HCT levels than the NC and PC pigs, while the MCV in GA pigs was higher than in NC and PC groups.

**Table 3:** Effect of postweaning enterotoxigenic *E. coli* (ETEC) F18 challenge and dietary supplementation with plant combinations on red blood cell, hemoglobin, hematocrit, and platelet concentrations in organic weaner pigs.

**Item^d^**	**Treatment^e^**				**SEM^f^**	***P*** **-value**		
	**NC**	**PC**	**GA**	**GB**		**Treat**	**Day**	**Treat** × **day**
RBC (10^6^/μL)						0.011	0.001	<0.001
Weaning	6.35	6.34	6.13	6.14	0.133			
Day 3	6.20	6.29	6.03	6.14	0.192			
Day 5	6.28	6.53	6.11	6.39	0.197			
Day 7	6.36^a^	7.29^b^	6.32^a^	6.27^a^	0.203			
Day 14	6.52	6.61	5.89	5.97	0.196			
Day 21	6.86^b^	6.75^b^	5.51^a^	5.83^a^	0.197			
HGB (g/L)						0.002	<0.001	<0.001
Weaning	114.02	114.15	114.86	110.11	1.982			
Day 3	111.63	117.37	112.75	112.00	3.781			
Day 5	113.87	118.09	114.04	113.73	2.730			
Day 7	117.63^a^	136.78^b^	121.24^a^	115.37^a^	3.328			
Day 14	123.98^b^	124.80^b^	115.10^a^	112.50^a^	2.701			
Day 21	129.98^b^	127.53^b^	114.93^a^	115.13^a^	2.873			
HCT (%)						0.029	<0.001	<0.001
Weaning	40.38^b^	39.61^ab^	39.92^ab^	37.58^a^	0.702			
Day 3	37.19	38.37	37.90	37.11	1.126			
Day 5	38.78	40.84	38.94	39.72	1.029			
Day 7	38.85^a^	44.44^b^	40.11^a^	38.34^a^	1.132			
Day 14	39.09	39.78	37.21	36.26	1.050			
Day 21	42.04^b^	41.62^b^	37.30^a^	37.10^a^	1.000			
MCV (fL)						0.112	<0.001	<0.001
Weaning	63.64	62.41	64.64	61.25	0.926			
Day 3	60.13	60.99	62.80	60.36	0.906			
Day 5	61.95	62.61	63.74	62.14	1.003			
Day 7	61.32	60.88	63.66	61.11	1.144			
Day 14	60.09	60.28	63.22	60.75	1.153			
Day 21	61.54^a^	61.49^a^	68.05^b^	63.91^ab^	1.453			
MCH (pg)						0.187	<0.001	<0.001
Weaning	17.97	18.03	18.63	17.94	0.254			
Day 3	18.04	18.68	18.70	18.23	0.442			
Day 5	18.19	18.00	18.69	17.80	0.253			
Day 7	18.57	18.71	19.06	18.42	0.291			
Day 14	19.07	18.87	19.46	18.89	0.306			
Day 21	19.01^a^	18.79^a^	20.86^b^	19.84^ab^	0.360			
MCHC (g/L)						0.896	<0.001	0.195
Weaning	282.58	288.09	287.21	292.50	3.701			
Day 3	300.25	305.75	297.88	301.75	3.314			
Day 5	293.87	289.81	292.60	285.18	3.545			
Day 7	303.06	306.49	302.46	301.25	3.715			
Day 14	317.06	313.52	308.26	310.87	3.506			
Day 21	308.77	305.82	306.59	310.50	3.557			
Reticulocytes (10^9^/L)						0.290	<0.001	<0.001
Weaning	209.53^b^	182.13^ab^	171.76^ab^	155.85^a^	13.609			
Day 3	152.65^c^	138.96^bc^	102.60^a^	112.86^ab^	11.647			
Day 5	182.21	153.76	115.46	119.28	18.099			
Day 7	201.78	163.81	166.59	177.35	24.218			
Day 14	172.99^a^	159.15^a^	360.49^b^	227.93^c^	29.300			
Day 21	173.31^a^	183.18^a^	418.74^c^	315.33^b^	19.697			
Platelets (10^9^/L)						0.207	0.103	0.706
Weaning	390.78	318.73	376.13	383.34	49.699			
Day 3	345.21	342.52	320.39	319.34	44.776			
Day 5	318.13	359.02	258.40	410.68	47.671			
Day 7	277.25	312.54	343.67	398.50	49.818			
Day 14	354.96	382.32	358.39	405.18	47.179			
Day 21	392.00	380.10	364.25	448.62	47.830			

abc Within a row, values that do not share a common superscript differ (*P* <0.05), Benjamini-Hochberg adjustment.

d RBC, Red blood cell count; HGB, Hemoglobin; HCT, Hematocrit; MCV, Mean cell volume; MCH, Mean Cell Hemoglobin; MCHC, Mean red blood cell hemoglobin content.

e NC, non-challenge (*n* = 8), standard diet; PC, challenged, standard diet (0–7 d, *n* = 8; 7–21 d, *n* = 7); GA, challenged, garlic + apple pomace (3 +3%; 0–7 d, *n* = 8; 7–21 d, *n* = 6); GB, challenged, garlic + blackcurrant (3 + 3%; *n* = 8).

f Pooled standard error of least squared means.

The white blood cell (WBC) count, and the neutrophil, and basophil proportions were not influenced by treatment (Table 4). The interaction between day and treatment influenced the proportion of lymphocytes, monocytes, and eosinophils. On the 1st day of the study, the monocytes proportion of the NC, GA, and GB groups was higher than that of the PC group. On day 3, the GA pigs had a higher proportion of monocytes than the NC pigs. On day 5, the GB group had a lower proportion of monocytes than the PC group. On day 21, the PC and GA groups had a lower proportion of lymphocytes than the NC group.

**Table 4:** Effect of postweaning enterotoxigenic *E. coli* (ETEC) F18 challenge and dietary supplementation with plant combinations on white blood cell differential of organic weaner pigs.

**Item^d^**	**Treatment^e^**				**SEM^f^**	***P*** **-value**		
	**NC**	**PC**	**GA**	**GB**		**Treat**	**Day**	**Treat** × **day**
WBC (10^9^/L)						0.702	0.185	0.419
Weaning	22.95	20.68	19.30	22.82	2.294						
Day 3	22.65	21.89	23.15	26.36	2.082						
Day 5	21.07	23.06	21.48	22.98	2.206						
Day 7	21.54	25.14	24.79	23.19	2.302						
Day 14	21.09	24.74	27.27	22.13	2.187						
Day 21	19.76	22.22	22.93	21.41	2.215						
Neutrophils (%)						0.361	0.035	0.271
Weaning	47.13	41.66	42.49	43.87	2.627						
Day 3	43.38	37.05	35.51	40.19	2.750						
Day 5	42.81	34.33	38.30	41.62	2.796						
Day 7	44.23	40.17	40.66	42.06	2.630						
Day 14	41.30	42.76	41.64	40.61	2.477						
Day 21	37.31	42.14	41.64	38.39	2.507						
Lymphocytes (%)						0.347	<0.001	0.033
Weaning	42.03	49.17	42.91	45.47	3.273						
Day 3	43.74	49.76	49.62	48.30	2.753						
Day 5	47.23	56.05	51.34	49.37	2.484						
Day 7	46.87	53.09	50.14	50.79	2.362						
Day 14	52.70	50.88	51.27	52.55	1.819						
Day 21	57.06^b^	50.91^a^	50.31^a^	55.08^ab^	1.680						
Monocytes (%)						0.012	<0.001	<0.001
Weaning	3.62^b^	1.13^a^	5.72^c^	4.87^bc^	0.642						
Day 3	4.61^a^	5.76^ab^	6.69^b^	4.66^a^	0.497						
Day 5	5.12^ab^	6.32^b^	5.90^ab^	4.54^a^	0.465						
Day 7	5.47	4.61	5.65	4.21	0.621						
Day 14	5.24	4.42	5.28	4.71	0.663						
Day 21	4.68	5.57	6.17	4.92	0.588						
Eosinophils (%)						0.767	<0.001	0.021
Weaning	7.57	10.04	8.90	6.14	1.217						
Day 3	8.27	7.36	8.16	6.81	1.170						
Day 5	4.81	3.36	4.36	4.58	0.708						
Day 7	3.30	1.92	3.65	2.91	0.569						
Day 14	1.07	1.87	1.63	2.07	0.479						
Day 21	1.09	2.00	1.83	1.61	0.483						
Basophils (%)						1.000	0.905	0.964
Weaning	0.05	0.03	0.10	0.04	0.312				
Day 3	0.00	0.06	0.01	0.03	0.156				
Day 5	0.02	0.03	0.01	0.03	0.194				
Day 7	0.01	0.01	0.00	0.02	0.113				
Day 14	0.00	0.01	0.07	0.05	0.203				
Day 21	0.03	0.03	0.05	0.00	0.182				

abc Within a row, values with different superscripts differ (*P* < 0.05), Benjamini-Hochberg adjustment.

abc Within a row, values that do not share a common superscript differ (*P* < 0.05), Benjamini-Hochberg adjustment.

d WBC, White blood cell count.

e NC, non-challenge (*n* = 8), standard diet; PC, challenged, standard diet (0–7 d, *n* = 8; 7–21 d, *n* = 7); GA, challenged, garlic + apple pomace (3 + 3%; 0–7 d, *n* = 8; 7–21 d, *n* = 6); GB, challenged, garlic + blackcurrant (3 + 3%; *n* = 8).

f Pooled standard error of least squared means.

### 3.5. Plasma acute phase proteins

The acute phase protein concentrations in plasma were influenced by the treatment and day (Table 5). The treatment effect on plasma pigMAP concentration was dependent on the day (*P* = 0.002). On day 7, the pigMAP concentration was lower in the NC and GA groups than in the PC group, whereas the GB group did not differ from the PC group. On day 21, the pigMAP concentration was higher in the GA, GB, and PC than in the NC group. The treatment effect on plasma CRP concentration was dependent on the day (*P* < 0.001). On the 1st day of the study, the GB group had lower levels of CRP compared to the other groups. On days 3 and 5, the GB pigs had a higher CRP concentration than the NC pigs. By the end of the study on day 21, the CRP concentration in the GA and GB pigs was higher than in the NC and PC pigs. The treatment effect on plasma haptoglobin concentration was dependent on the day (*P* < 0.001). On day 5, the haptoglobin concentration in the NC, GA and GB pigs did not differ from the PC group but it was higher in the GB than in the NC pigs. On day 7, the haptoglobin concentration in the NC and GB pigs did not differ from the PC group, whereas that of the GA group was lower than the PC and GB groups. By the end of the study on day 21, the haptoglobin concentration of the NC, GA and GB pigs was lower than the PC pigs.

**Table 5:** Effect of postweaning enterotoxigenic *E. coli* (ETEC) F18 challenge and dietary supplementation with plant combinations on pig major acute protein (pigMAP), C-reactive protein (CRP), and haptoglobin (HPT) levels in plasma of organic weaner pigs.

**Item**	**Treatment^d^**				**SEM^e^**	***P*** **-value**		
	**NC**	**PC**	**GA**	**GB**		**Treat**	**Day**	**Treat** × **day**
pigMAP (mg/L)						0.151	<0.001	0.002
Weaning	751.96	759.17	786.98	739.83	63.882			
Day 3	733.24	774.91	792.57	790.87	60.513			
Day 5	669.56	805.78	876.11	846.53	71.337			
Day 7	780.51^a^	1055.81^b^	770.71^a^	852.91^ab^	63.282			
Day 14	634.03	777.25	677.80	711.84	91.095			
Day 21	353.63^a^	469.95^b^	522.48^b^	504.33^b^	32.603			
CRP (mg/L)						0.044	<0.001	<0.001
Weaning	19.70^b^	19.16^b^	21.45^b^	11.44^a^	3.632			
Day 3	15.18^a^	22.47^ab^	17.51^ab^	26.51^b^	4.565			
Day 5	16.16^a^	22.51^ab^	19.65^ab^	29.63^b^	4.711			
Day 7	24.41	19.86	16.08	28.01	5.455			
Day 14	12.65	8.59	19.10	20.13	4.444			
Day 21	4.16^a^	6.91^a^	17.88^b^	13.34^b^	2.841			
HPT (mg/mL)						0.002	<0.001	<0.001
Weaning	1.26	1.14	1.22	1.12	0.102			
Day 3	1.65	1.92	1.92	2.00	0.165			
Day 5	1.89^a^	2.49^ab^	2.12^ab^	2.71^b^	0.214			
Day 7	2.26^ab^	2.85^b^	1.83^a^	2.80^b^	0.271			
Day 14	1.54	1.66	1.41	2.19	0.242			
Day 21	0.86^b^	1.60^c^	0.26^a^	0.55^b^	0.165			

abc Within a row, values that do not share a common superscript differ (P <0.05), Benjamini-Hochberg adjustment.

d NC, non-challenge (n = 8), standard diet; PC, challenged, standard diet (0–7 d, *n* = 8; 7–21 d, *n* = 7); GA, challenged, garlic + apple pomace (3 + 3%; 0–7 d, *n* = 8; 7–21 d, *n* = 6); GB, challenged, garlic + blackcurrant (3 + 3%; *n* = 8).

e Pooled standard error of least squared means.

### 3.6. 16S rRNA gene sequencing

A total of 147 fecal samples were sequenced. An average of 1,047 ASVs were identified per sample coming from 9,572,592 reads. On average, 78% of the identified ASVs were retained following size filtering, corresponding to 98.5% of the high-quality reads (Supplementary Table 3).

### 3.6.1. Alpha-diversity

**Figure 6:**
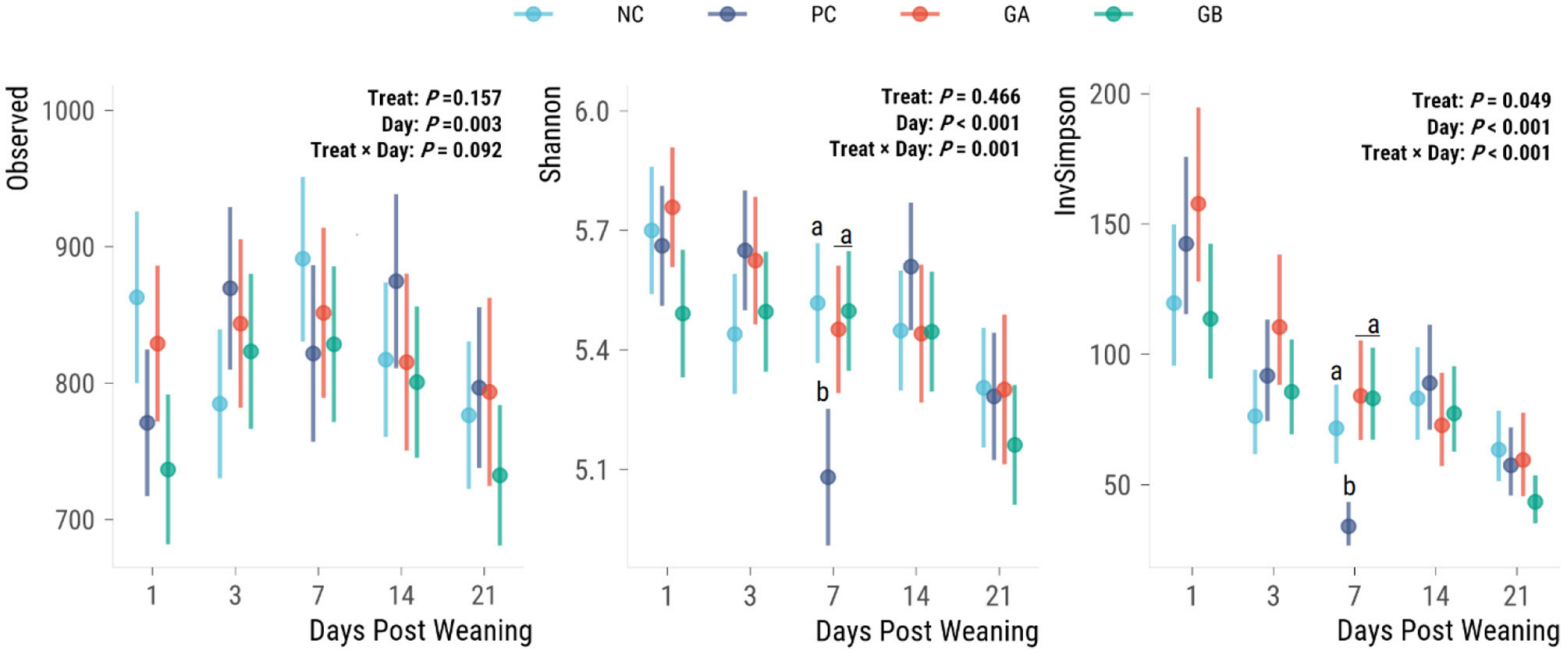
Effect of postweaning enterotoxigenic *E. coli* (ETEC) F18 challenge and supplementation with plant combinations on α-diversity measures of the fecal microbiota. Dots are least squared means and lines are 95% confidence intervals. The ETEC F18 was administered orally on days 1 and 2. ^a,b^Values that do not share a common superscript differ (*P* < 0.05), Benjamini-Hochberg adjustment. NC, non-challenge, standard diet (*n* = 8); PC, challenged, standard diet (0–7 d, *n* = 8; 7–21 d, *n* = 7); GA, challenged, garlic + apple pomace (3 + 3%; 0–7 d, *n* = 8; 7–21 d, *n* = 6); GB, challenged, garlic + blackcurrant (3 + 3%; *n* = 8).

There was no effect of treatment on the observed α-diversity (Figure 6), whereas the treatment effect on Shannon (*P* = 0.002) and Inverse Simpson (*P* < 0.001) indices were dependent on the day. Observed α-diversity generally increased from days 1 to 7 and then decreased again on day 21. On day 7, the GA, GB, and NC pigs had a greater Shannon and inverse Simpson α-diversity index than the PC pigs.

#### 3.6.2. Beta-diversity

**Figure 7:**
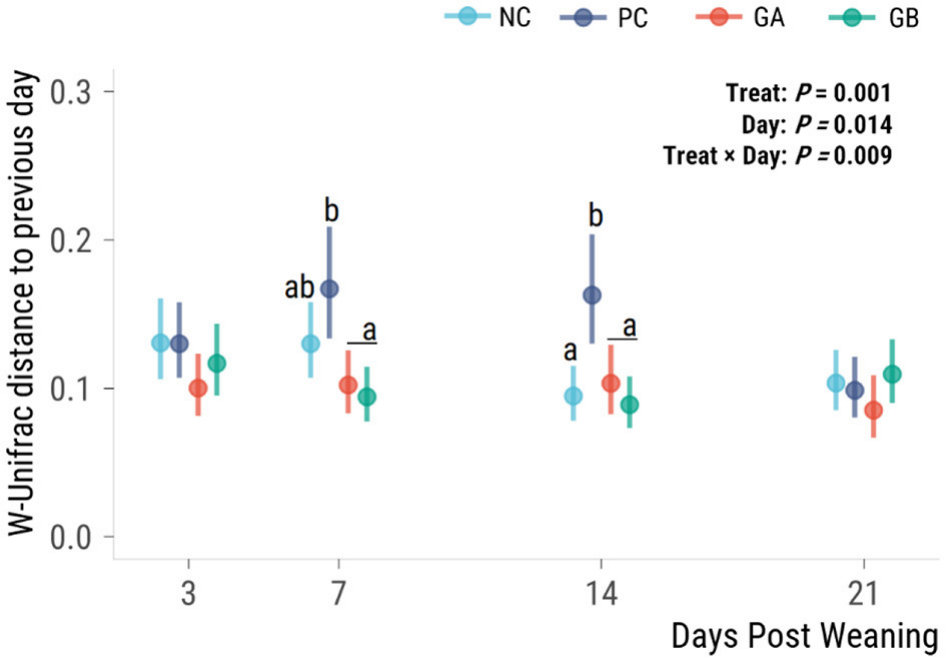
Effect of postweaning enterotoxigenic *E. coli* (ETEC) F18 challenge and supplementation with plant combinations on β-diversity measures of the fecal microbiota. Weighted Unifrac distance difference from the prior time point. Dots are least squared means and lines are 95% confidence intervals. The ETEC F18 was administered orally on days 1 and 2. ^a,b^Values that do not share a common superscript differ (*P* < 0.05), Benjamini-Hochberg adjustment. NC, non-challenge, standard diet (*n* = 8); PC, challenged, standard diet (0–7 d, *n* = 8; 7–21 d, *n* = 7); GA, challenged, garlic + apple pomace (3 + 3%; 0–7 d, *n* = 8; 7–21 d, *n* = 6); GB, challenged, garlic + blackcurrant (3 + 3%; *n* = 8).

The effect of treatment on the volatility (distance from the previous observation of individual community structures, i.e., indicator of stability over time) of the fecal microbiota (*P* = 0.009) was dependent on the day (Figure 7). The PC group had a greater microbiota volatility than all other groups on days 7 and 14. No significant difference in the distance from the previous day was observed on days 3 or 21.

Treatments influenced β-diversity of the fecal microbiota (Supplementary Figure 1). On day 1, the GA pigs resembled the NC pigs, while the rest differed. On day 3, the β-diversity of the PC group differed from that of NC, whereas that of the GA and GB groups were similar to that of the NC pigs. On day 7, all groups differed in terms of β-diversity, but the GA and GB groups appeared to cluster closer to the NC piglets. By day 14, the PC group was similar to the NC pigs but differed from the GA and GB groups, and the GA group also differed from the NC. All groups were different by day 21. On days 14 and 21, the GA and GB distances were less dispersed than in the NC and PC groups.

#### 3.6.3. Relative abundance and differential abundance

**Figure 8:**
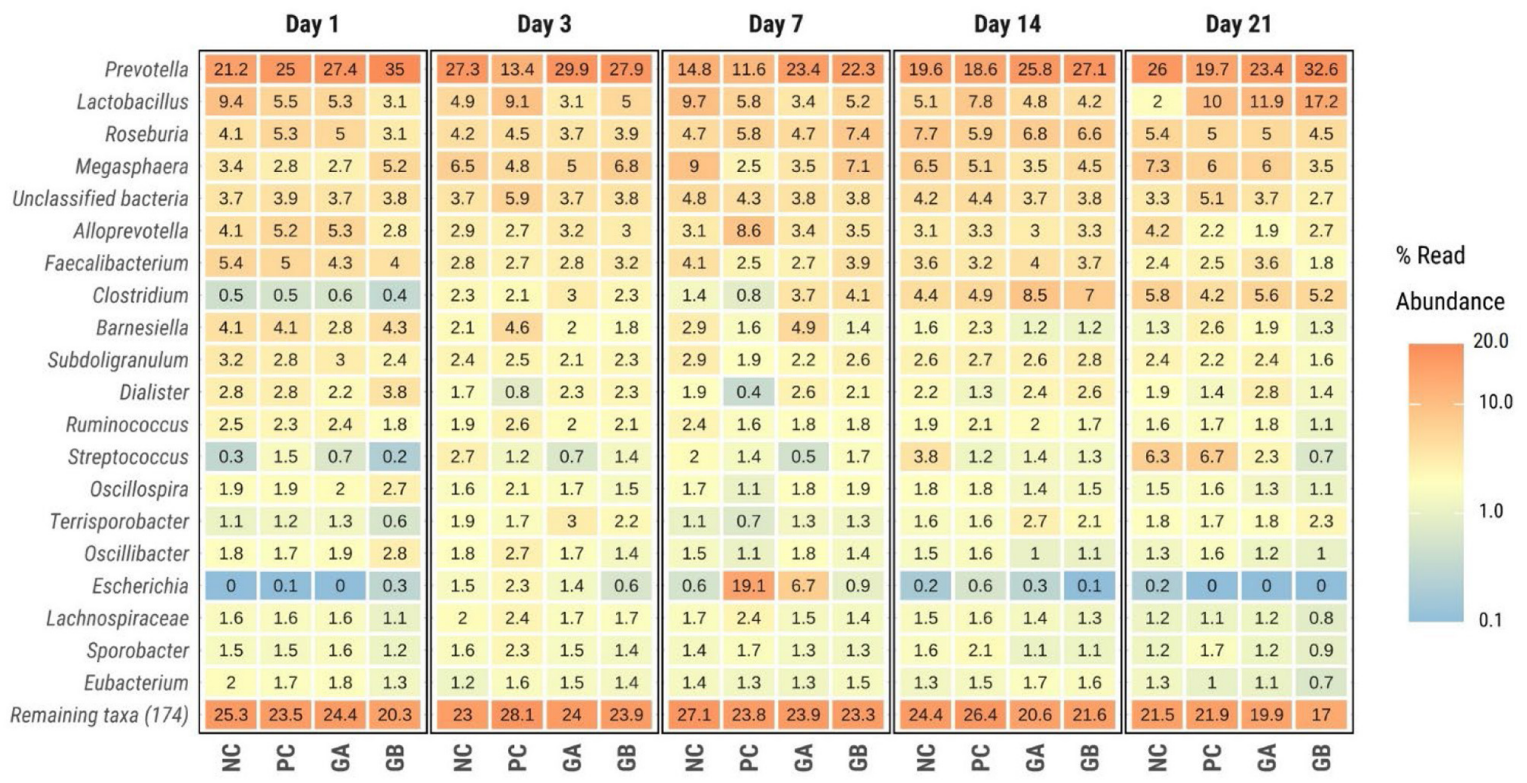
Relative abundance of bacterial genera within the feces of pigs challenged with enterotoxigenic *E. coli* (ETEC) F18 after weaning and supplemented with plant combinations. The ETEC F18 was administered orally on days 1 and 2. Values indicate mean relative abundance in percentage of the 20 dominant genera (Y-axis) across treatments (X-axis) and days postweaning (panel). NC, non-challenge, standard diet (*n* = 8); PC, challenged, standard diet (0–7 d, *n* = 8; 7–21 d, *n* = 7); GA, challenged, garlic + apple pomace (3 + 3%; 0–7 d, *n* = 8; 7–21 d, *n* = 6); GB, challenged, garlic + blackcurrant (3 + 3%; *n* = 8).

Firmicutes (>48%) and Bacteroidetes (>27%) were the most dominant phyla in the feces of all treatment groups throughout the study (Supplementary Figure 2). For most days, the predominant genera were *Prevotella, Lactobacillus, Roseburia*, and *Megasphera* (Figure 8). *Prevotella* was the most abundant (>13%) on most days and in most groups, except for the PC pigs on day 7, where *Escherichia* dominated (19.1%).

**Figure 9:**
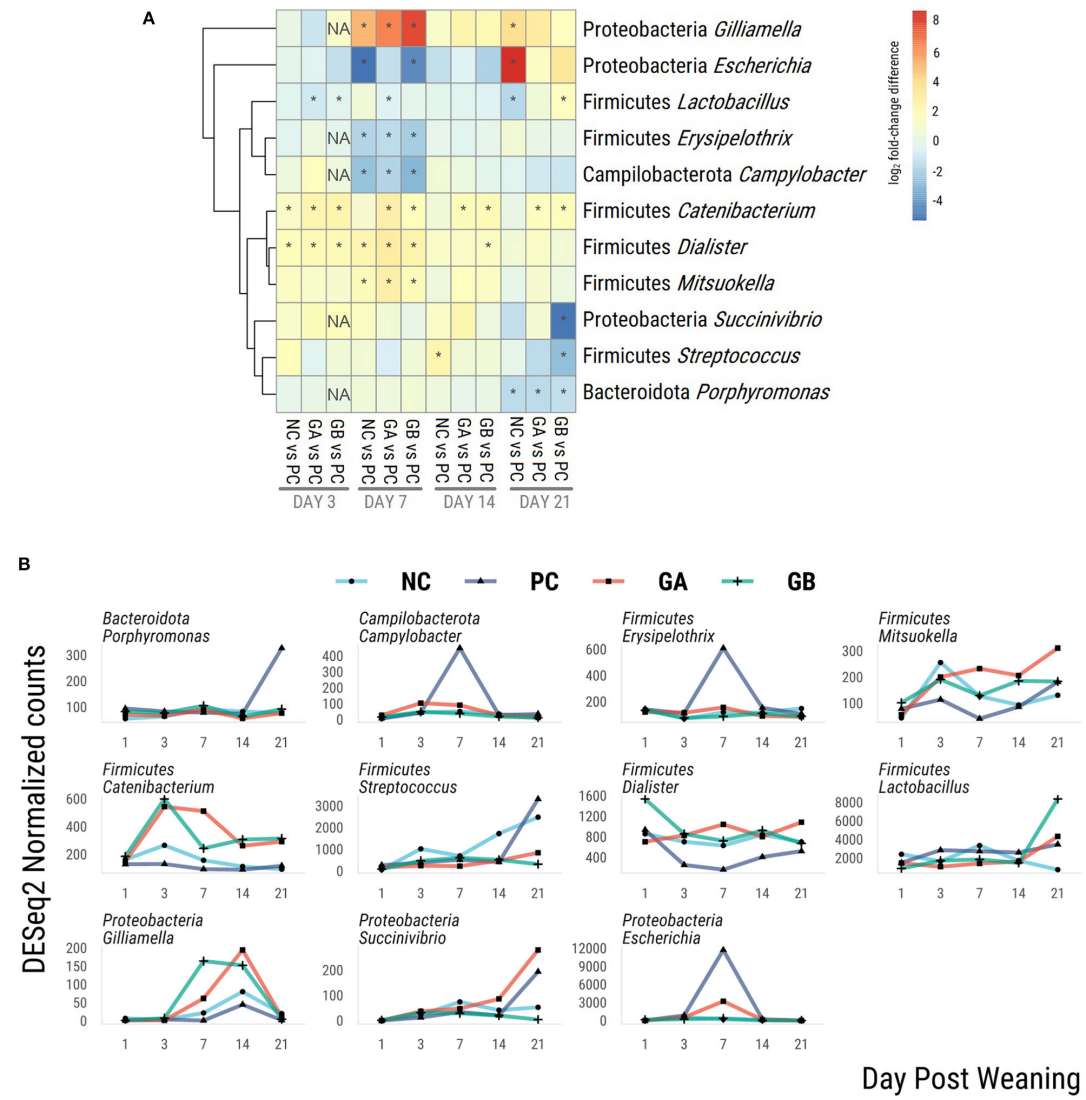
Differential abundance of bacterial genera within the feces of pigs challenged with enterotoxigenic *E. coli* (ETEC) F18 after weaning and supplemented with plant combinations. The ETEC F18 was administered orally on days 1 and 2. **(A)** log_2_-fold-change difference from PC. *adj-*P* < 0.05 pairwise comparison against PC within day. NA, *P* value no estimable. **(B)** DESeq2 normalized counts of differentially abundant genera. Only differentially abundant taxa (log_2_FC > 2; *P* < 0.05) are represented in the figures. NC, non-challenge, standard diet (*n* = 8); PC, challenged, standard diet (0–7 d, *n* = 8; 7–21 d, *n* = 7); GA, challenged, garlic + apple pomace (3 + 3%; 0–7 d, *n* = 8; 7–21 d, *n* = 6); GB, challenged, garlic + blackcurrant (3 + 3%; *n* = 8).

Eleven genera were identified as differentially abundant (threshold: log_2_FC > 2, *P* < 0.05) in at least one group at a given time when compared against the PC group (Figure 9). The hierarchical dendrogram clustered *Catenibacterium, Dialister, and Mitsoukella* as generally more abundant in the NC, GB, and GA groups than in the PC group, whereas *Lactobacillus, Erysipelonthrix*, and *Campilobacter* clustered as generally less abundant.

On day 7 postweaning, *Gilliamella* was more abundant in NC, GA, and GB than in the PC group. *Escherichia* was less abundant in the NC and GB groups, and *Campylobacter* and *Erysipelothix* were less abundant in NC, GA, and GB than in PC on day 7. Compared to the PC group, *Lactobacillus* was less abundant in the NC group on day 21, in the GA group on days 3 and 7, and in the GB group on day 3. *Catenibacterium* was more abundant in the NC group on day 3, and consistently more abundant in the GA and GB than in the PC pigs. *Dialister* was more abundant in the NC group on days 3 and 7, and in the GA and GB groups on days 3, 7, and 14 than in the PC. *Mitsoukella* was more abundant in the NC, GA, and GB than in the PC pigs on day 3. *Succinivibrio* was less abundant only in the GB pigs on day 21.

Forty-two species were identified as differentially abundant (Supplementary Figure 3). The species that followed a similar behavior to that observed at genus level were: *E. coli, Campylobacter fetus, L. johnsonii, L. delbrueckii, L. pontis, L. amylovorus, L. reuteri*, and *Catenibacterium mitsoukai*.

## 4. Discussion

Diarrhea occurrence and bacterial fecal shedding are part of the evaluation of ETEC challenge models for PWD (19). The current study found signs of ETEC infection in the PC pigs (but not in the NC), indicating the validity of the ETEC challenge model. Limited reports exist on ETEC challenge models with organic weaner pigs. In the study of Sørensen et al. (39), pigs were weaned at a similar age as in the current study, and the authors noted difficulties in using the ETEC challenge model to consistently introduce a diarrhea-like state. Hedemann and Bach Knudsen (40) observed low F-DM during 2 days with peaks on days 4 and 5 postweaning (1–2 days postinfection) with pigs weaned at 7 weeks following an ETEC F4 challenge. Our study was able to induce a diarrhea-like state in the PC pigs for ≈5 days consistently differing from the NC group. This was despite the relatively high weaning age and weight of the animals, both of which have been reported as factors that could affect the efficacy of the challenge model (19). The challenge dosage in the current study was higher than that of the two aforementioned studies (10^9^ vs. 10^8^ CFU/day), which could have been a factor determining the effectiveness of the challenge model in the present study.

All pigs started the study in good health. On arrival, ETEC F18 and the virulence gene *fedA* (encoding for the F18 fimbriae) were not detectable, whereas pigs shed detectable levels of the *est*-*II* gene encoding for the STb toxin. Similar observations have been reported in previous ETEC challenge studies. Spitzer et al. (41) reported the presence of the *est-II* gene but not the F4 encoding gene in the feces of healthy weaned pigs prior to ETEC F4 challenge. Rhouma et al. (42) similarly found genes encoding STa and STb (but not F18 or F4) in the feces of piglets before oral ETEC F4 challenge. Since the piglets did not have diarrhea on arrival, the findings indicate that the fecal presence of *E. coli* positive for toxins but negative for F4 or F18 fimbriae may not always be associated with diarrheal symptoms.

During the 1st week postweaning, the PC pigs displayed increased fecal shedding of ETEC F18 and virulence factor genes, higher fecal scores (more liquid feces), lower F-DM, and decreased growth metrics, which coincide with typical signs of PWD in infection models (19, 43). Furthermore, on day 7 postweaning, the RBC, HGB, and HCT levels were elevated in the PC pigs, with HGB and HCT levels exceeding reference intervals for their age (44, 45). The elevated levels of RBC, HGB, and HCT (on day 7) in the PC pigs could be linked to dehydration, which corresponds to the diarrheal symptoms (2) observed during the 1st week after weaning (5–6 days postinfection).

We observed increased levels of reticulocytes (immature erythrocytes) near the end of the study, a relatively lower RBC, HGB, and HCT, higher MCV, and no change in MCHC in the GA and GB pigs compared to the PC and NC pigs. This is noteworthy because garlic constituents can cause oxidative hemolysis (46) and excessive intake can result in anemia (47). In the current study, although the RBC were slightly below reference levels, the HGB, HCT, MCV, and MCHC were not abnormal, indicating that the pigs were not anemic. This state, in which garlic induces mild chronic hemolysis without resulting in anemia, has been proposed as a potential mechanism involved in the antiplatelet, pro-circulatory, anti-inflammatory, and antiapoptotic effects associated with the therapeutic use of garlic products (48). According to Akgül et al. (48), the health benefits of garlic consumption could be a manifestation of achieving homeostasis, partly mediated by younger RBC. However, because the study was conducted in mice (48) and there are no follow-up studies, more research on these mechanisms and their relevance for other species is required.

Different types of pathologies and inflammation processes can result in variable kinetic patterns for pigMAP, CRP, haptoglobin, and other acute phase proteins. Moreover, monocytes and particularly neutrophils are expected to play an effector role in response to bacterial infections. The pigMAP, haptoglobin, and monocyte levels followed a similar pattern in all groups in the current study, rising during the 1st week, peaking on days 5 or 7, and then declining (except for monocytes in GB pigs). However, no significant response was observed in the other white blood cell pools, and the concentration of monocytes and neutrophils were within normal limits (44, 45). The weaning process has been shown to cause transient inflammation in piglets that upregulates gut pro-inflammatory cytokines (49). Similarly, elevated levels of pigMAP and haptoglobin have been observed in ETEC-challenged pigs (50, 51). Hence both, weaning-related stress and ETEC challenge, could have contributed to the acute phase response observed under the current experimental conditions. Clapperton et al. (52) documented the positive association between pigMAP, haptoglobin, and monocyte concentrations, which we also observed in the current study. This might be due to activated monocytes producing pro-inflammatory cytokines, particularly interleukin 6, which stimulates hepatic production of pigMAP and haptoglobin.

On days when the PC pigs had acute PWD symptoms (days 5–9 postweaning), the GA and GB pigs had lower fecal scores (more solid feces), higher fecal dry matter, and lower fecal shedding of ETEC F18 bacteria (and associated virulence factors), indicating that GA and GB supplementation provided protection against ETEC-induced diarrhea. It is worth noting that, when compared to the PC group, the pigs in the GA and GB groups shed comparable amounts of ETEC and virulence factors during the first 3 days postinfection, indicating that the GA and GB piglets were infected with ETEC but did not develop PWD. There are no previous reports on the use of GA or GB supplementation in pigs. Dietary supplementation with garlic has been of interest and there are reports in the literature showing increased growth performance (although not always consistent), lower diarrhea incidence, and reduced *E. coli* shedding (53). However, there are few studies evaluating the effectiveness of garlic supplementation under controlled ETEC infection settings. Liu et al. (54) reported reduced serum haptoglobin concentrations, lower fecal scores and less diarrhea frequency in ETEC-challenged pigs fed a diet containing 10 ppm of garlic botanical (extract standardized to contain 40% propyl thiosulfonates), but no change was observed in ETEC fecal shedding. We similarly observed reduced diarrheal symptoms in pigs but accompanied by a reduced ETEC shedding. This point to the antibacterial effect against ETEC of the supplementation used in the current study, in contrast with Liu et al. (54) where antidiarrheal properties were attributed to immunomodulation.

The antibacterial effect observed here may be due to the higher inclusion of garlic, the preparation of the powder (11) or the synergistic action of garlic combined with the acidifying properties of apple pomace and blackcurrant. Allicin from garlic inhibits the growth of *E. coli* by inducing thiol stress and decreasing S-allylmercapto modification of proteins and glutathione pool, resulting in a total reduction in sulfhydryl level (55). The garlic cultivar and the processing method of the garlic powder can affect the potential content of allicin. Therador cultivar was identified as a high-allicin cultivar among over 10 garlic cultivars tested (Jensen, M., personal communication), and the preparation of the powder warranted allicin formation. The acidifying properties of apple pomace and blackcurrant may also prevent pathogen overgrowth because some pathogenic bacteria, including *E. coli*, are susceptible to low pH exposure (7). Indeed, organic acids have been used in piglet diets as antimicrobial agents and proven effective by providing an unfavorable environment for pathogenic bacteria and/or direct bactericidal action (56). Furthermore, the combination of organic acids with phytochemical compounds may synergistically contribute to productive performance reduced ETEC adherence to intestinal epithelium and overall enhanced intestinal health (57). The efficacy of the plant combinations was tested exclusively against ETEC F18 in this study. However, based on the proposed mode of action and our previous *in vitro* studies testing the antibacterial impact of similar plant materials against ETEC F4 (9), it is plausible that the combinations used in the current study may exhibit *in vivo* bactericidal activity against other strains, e.g., ETEC F4.

Because there are no reports in the literature on the composition of the fecal microbiota of ETEC-challenged organically raised weaned pigs, the current study serves as a reference for future research. We used three α-diversity measures to evaluate the effects of GA and GB supplementation on the richness and evenness of the microbiota of ETEC-challenged piglets. Reduced α-diversity (Shannon and Inverse Simpson indices) of the fecal microbiota of PC pigs were observed on day 7 postweaning. This could be a sign of dysbiosis, which is characterized by decreased microbial diversity (58). A clear decrease in α-diversity following diarrheal events has been observed in human infants (59), and some authors have also observed decreased α-diversity in fecal microbiota of diarrheal piglets (58). However, other studies evaluating ETEC challenge effects on fecal microbiota have failed to observe significant differences in α-diversity (60–62). In agreement with our findings, Rhouma et al. (63) reported reduced α-diversity in the fecal microbiota of pigs challenged with ETEC-F4, which was coincided with acute PWD symptoms, as opposed to other studies that only achieved subclinical states. Similarly, we also observed acute PWD symptoms in the PC group, emulating field conditions of the disease (2). The supplementation of GA and GB prevented a decrease in the richness and evenness of the fecal microbiota of piglets challenged with ETEC. Dietary supplementation with garlic has been reported to increase α-diversity of the fecal microbiota of mice (64) and sows (65). Apple pomace supplementation has also been shown to increase the α-diversity of the cecal mucosa of pigs (66). Interestingly, blackcurrant supplementation showed no effect on α-diversity in broilers (67). A highly diverse microbiota is usually regarded as beneficial to the host, and reduced diversity is associated with acute and chronic diseases (68). The use of antibiotics against ETEC infection often results in reduced diversity of the gut microbiota (58, 63), which in long run may give the opportunity for pathogens to colonize and cause diseases (58). Thus, the effect of GA or GB supplementation on α-diversity under ETEC challenge conditions can be seen as a positive outcome and is an indication of the targeted nature of the strategy.

We used weighted UniFrac distances to address the degree of differences in fecal microbiota because it considers phylogenetic and abundance information. Additionally, we investigated how the individual microbiota structure changes over time (in relation to the previous observation), known as microbiota volatility (69), as opposed to stability. In this study, we observed increased volatility of the microbiota by day 7 in both ETEC-challenged and unchallenged pigs, and only in the PC group on day 14. This is consistent with previous studies indicating that weaning is associated with abrupt changes in the gut microbiota (70). Furthermore, increased volatility of the gut microbiota has been proposed as a defining feature of (disease-related) dysbiosis (71). Increased volatility has also been linked to stress in mice and humans (31). We found that the ETEC F18 challenge delayed the stabilization of the fecal microbiota after weaning, as observed by the increased volatility; however, this was prevented with GA or GB supplementation.

Throughout the study, the ETEC F18 challenge influenced the microbial community structure (β-diversity) of pigs. Although PWD signs had disappeared in the PC group 2 weeks after weaning, the fecal microbiota composition remained different from that of the NC pigs. Rhouma et al. (63) observed a significant effect of ETEC challenge on β-diversity, even up to 5 weeks after weaning, indicating that the PWD episodes had a long-term impact on piglet fecal microbiota. In the current study, following the ETEC F18 challenge, the microbiota configuration of pigs in the GA and GB groups differed from the PC pigs consistently. Similar effects have been observed 40 days postweaning in pigs supplemented with pharmacological levels of zinc oxide during the nursery period (72), and 21 days postweaning following chito-oligosaccharide supplementation to piglets during an ETEC challenge (73). We only followed the pigs up to 3 weeks after weaning, but considering the aforementioned studies, a lasting effect of GA and GB supplementation on the microbiota structure of pigs may be expected.

During this study, the most abundant phyla within the fecal microbiota were Firmicutes and Bacteroidetes, which agrees with previous reports in weaned piglets (70). The ETEC challenge, as expected, increased the relative abundance of Proteobacteria due to an increase in *E. coli*, particularly during the 1st week postweaning. This in turn coincided with the PWD symptoms and fecal ETEC shedding in the PC pigs. The increased abundance of the *Escherichia* also coincided with the increased abundance of *Campylobacter, Erysipelothrix*, and lower abundance of *Catenibacterium, Dialister*, and *Mitsoukella*. Previous research has shown an increased abundance of *Campylobacter* after weaning, which is regarded as an opportunistic pathogen and is thought to be linked to the occurrence of PWD (74, 75). *Erysipelothrix* have also been shown to cause disease, particularly in older pigs after the decline of maternal antibodies (76). On the other hand, *Dialister* has been shown to be strongly associated with *Prevotella* and some blood parameters (positively with monocytes, platelets and negatively with eosinophils, and red blood cell parameters) (77). Smith et al. (78) also observed decreased abundance of *Dialister* and *Catenibacterium* following ETEC F18 challenge. *Mitsuokella* has been positively associated with body weight, along with *Prevotella* (79). In agreement with our findings, Duarte et al. (80) also reported a reduced abundance of *Mitsuokella* in response to ETEC F18 challenge. *Mitsuokella* has been shown to be more abundant in pigs fed diets supplemented with bacitracin and challenged with ETEC F18 (81). Thus, the ETEC challenge was associated with an increase in the abundance of potentially pathogenic bacteria at the expense of commensal bacteria.

Supplementation with GB prevented the overgrowth of *E. coli* after challenge, whereas the difference between GA and PC pigs was not significant. *Catenibacterium mitsuokai* was consistently more abundant in the GA and GB groups than in the PC. This genus is a Gram-positive anaerobe that produces acetic, lactic, butyric and iso-butyric acids from glucose (82). *Catenibacterium* has previously been reported as exclusively present in pigs supplemented with inulin, when compared to others receiving different dietary fiber types (83) and when supplementing with probiotics (84). Others have also shown that apple pomace supplementation increases the abundance of *Catenibacterium* in piglets (66). However, there is no reported evidence linking garlic or blackcurrant supplementation to *Catenibacterium*. This suggests that *Catenibacterium* abundance may be related to the dietary fiber or polyphenols in GA and GB diets. We observed an increased abundance of several species of *Lactobacillus* in the GB group toward the end of the study. *Lactobacillus* is well-known to promote immune cell homeostasis and intestinal health in the host. In agreement with our findings, feeding rats a diet supplemented with blackcurrant extract resulted in a significant increase in the cecal counts of lactobacilli (85). Aged garlic extract supplementation in humans resulted in increased fecal microbial richness, with a significant increase in *Lactobacillus* and *Clostridia* abundance (86). In contrast, Colombino et al. (67) observed reduced abundance of *Lactobacillus* in the excreta of broilers fed blackcurrant pomace. The increased abundance of *Lactobacillus* in our study and others might be related to a prebiotic effect of garlic and blackcurrant. The GB group also had a consistently higher abundance of *Prevotella copri*. *Prevotella* is expected to increase after weaning and to remain more abundant in piglets during the nursery and growing stages (87). The evidence regarding the association between *Prevotella* and diarrhea has been contradictory, but it appears that animals with higher *Prevotella* abundance may have better protection against diarrhea (87). *Prevotella* is widely associated with plant polysaccharide consumption (88), thus comparative advantage for these substrates might have contributed to the higher abundance in the GB group.

The plant inclusion level in the feed was based on expected concentration in the stomach and confirmed *in vitro* bactericidal activity in porcine gastric digesta, with no observed adverse effects on feed intake or toxicity. A higher dose may be more effective, but limitations regarding toxicity, diet formulation, and economics need to be considered. Both the GA and the GB were successful in preventing ETEC (and other potentially pathogenic bacteria) proliferation, thereby preventing PWD development. Furthermore, GA and GB had no adverse effect on piglet fecal microbiota, favoring the abundance of commensal bacteria. Nevertheless, it appeared that GB supplementation was more effective than GA. We observed that the pH of the blackcurrant powder (2.81 ± 0.01) was lower than that of the apple pomace powder (3.28 ± 0.01) and the ABC-4 of the GB diet was lower than that of the GA diet. Apples harvested before maturity normally have a higher content of organic acids and display lower pH than mature apples and thus unripe apples may be a resource to study more in future. Ascorbic acid and anthocyanins from blackcurrant have shown high stability following simulated gastric digestion (89). The higher acidifying properties of the GB treatment may have therefore contributed to the greater effectiveness observed. However, other mechanisms also seem to play a role, particularly with regard to microbiota modulation. Esposito et al. (90) reported, for example, that blackcurrant anthocyanins have an anti-inflammatory effect that may be mediated by interaction with the local microbiota and their functions in the intestine. The dietary fiber fraction may also have an impact on the gut microbiome, particularly the fermentative species, and thus on the production of lactic acid and short-chain fatty acids, which may further lower the pH locally (57). However, the dietary fiber profiles of the experimental diets used in this study were similar, except for a higher fructan content in GA and GB diets. Thus, more research is required to investigate the roles of dietary fiber and bioactive compounds in GB (e.g., anthocyanins), as well as potential interactions with the gut microbiota.

## 5. Conclusion

In conclusion, the ETEC F18 challenge as conducted here resulted in decreased growth, evident signs of PWD, and increased abundance of ETEC and other harmful bacteria in the feces of organically raised piglets weaned at 7 weeks of age. The weaning process and the ETEC infection induced high levels of acute phase response parameters during the 1st week postweaning. Dietary GA and GB supplementation offered protection against PWD caused by ETEC F18 infection, inhibiting bacterial pathogen proliferation while maintaining fecal microbiota diversity, reducing volatility of the microbiota structure, and favoring the abundance of beneficial bacteria. The use of GA and GB provides a safe nutritional strategy for management of postweaning diarrhea caused by *E. coli* F18 infection and possibly other ETEC strains. Thereby reducing the need for veterinary medical interventions in organic- and possibly conventional pig production.

Further research using multi-omics approaches may be useful to further investigate the mode of action of GA and GB supplementation and the modulation of gut microbiome, as well as the effects on oxidative capacity and immunological regulation. Further research into the role of GA or GB supplementation in protection against other pathogenic infections is warranted. Furthermore, the long-term effects of postweaning GA or GB supplementation would be relevant to investigate.

## Data availability statement

The data presented in the study are deposited in the European Nucleotide Archive (ENA; https://www.ebi.ac.uk/ena/browser/home), accession number PRJEB57915.

## Ethics statement

The animal study was reviewed and approved by Danish Animal Experiments Inspectorate Ministry of Food, Agriculture and Fisheries Danish Veterinary and Food Administration License 2017-15-0201-01270.

## Author contributions

MJ, NC, and OH contributed to conception and design of the study. KJB, NC, and PC curated the data. KJB and PC contributed to the bioinformatic and statistical analyses. MJ, NC, OH, PL, GG, and PC provided scientific input and edited the manuscript. KJB wrote the first draft of the manuscript. All authors contributed to manuscript revision and approved the submitted version.
