# Supplementary material for: Antibacterial plant combinations prevent postweaning diarrhea in organically raised piglets challenged with enterotoxigenic Escherichia coli F18
Source: Front Vet Sci. 2023 Apr 3;10:1095160. doi: 10.3389/fvets.2023.1095160 (PMC10106643; doi:10.3389/fvets.2023.1095160)
Supplement: Supplementary file 1 [file Data_Sheet_1.pdf]

Antibacterial plant combinations prevent postweaning diarrhea in organically raised piglets challenged with enterotoxigenic *Escherichia coli* F18

Supplementary Material

Table S1 Ingredient composition of the experimental diets (as-fed basis, %)

Item, %	Diets[1]		
	NC - PC	GA	GB
Organic wheat	25.75	25.75	25.75
Barley	22.25	22.25	22.25
Organic oats	18.60	12.60	12.60
Garlic powder	0	3.00	3.00
Apple Pulp powder	0	3.00	0
Blackcurrant powder	0	0	3.00
Fishmeal	6.00	6.00	6.00
Organic soy cake	5.70	5.70	5.70
Horse beans	5.00	5.00	5.00
Organic rye	5.00	5.00	5.00
Potato protein	4.40	4.40	4.40
Organic barley	2.80	2.80	2.80
Organic wheat bran	2.00	2.00	2.00
Calcium carbonate	1.15	1.15	1.15
Monocalcium phosphate	0.47	0.47	0.47
Vitamin + Mineral premix[2]	0.40	0.40	0.40
Vitamin E	0.2	0.2	0.2
NaCl	0.28	0.28	0.28

[1] NC: non-challenge, standard diet; PC: challenged, standard diet; GA: challenged, Garlic and Apple pomace supplementation (3%+3%); GB: challenged, garlic and blackcurrant supplementation (3%+3%).
[2] Provided per kg of diet: 173 mg Fe (iron sulfate), 80 mg Cu (copper sulfate), 80 mg Cu (copper sulfate), 46 mg Mn (manganese oxide), 100 mg Zn (Zinc oxide), 0.30 mg I (calcium iodate), 0.30 mg Se (sodium selenite), 5400 UI vitamin A, 1000 IU vitamin D3, 215 IU vitamin E.

Table S2 Quantitative PCR primer details and assay settings

Primer name	Target sequence	Sequence (5'-3')	Conc[1] (mM)	T_A[2] (°C)	Size[3] (bp)
F18 F	*E. coli* F18 fimbriae (*FedA*)	GGAGGTTAAGGCGTCGAATAG	0.3	62	90
F18 R		CCACCTTTCAGTTGAGCAGTA	0.3		
STb F	*E. coli* STb toxin (*estB*)	TGCCTATGCATCTACACAAT	0.3	59.1	113
STb R		CTCCAGCAGTACCATCTCTA	0.3		

[1]Concentration in qPCR reactions
[2]Annealing temperature
[3]Amplicon size

Table S3 DADA2 read tracking summary

Item	Average
Original (ASVs)	1,047.33
Original (read counts)	65,119.67
Low abundance removed ASV	822.92
Low abundance removed read counts	64,161.07
ASVs retained (%)	78.70
Reads retained (%)	98.52

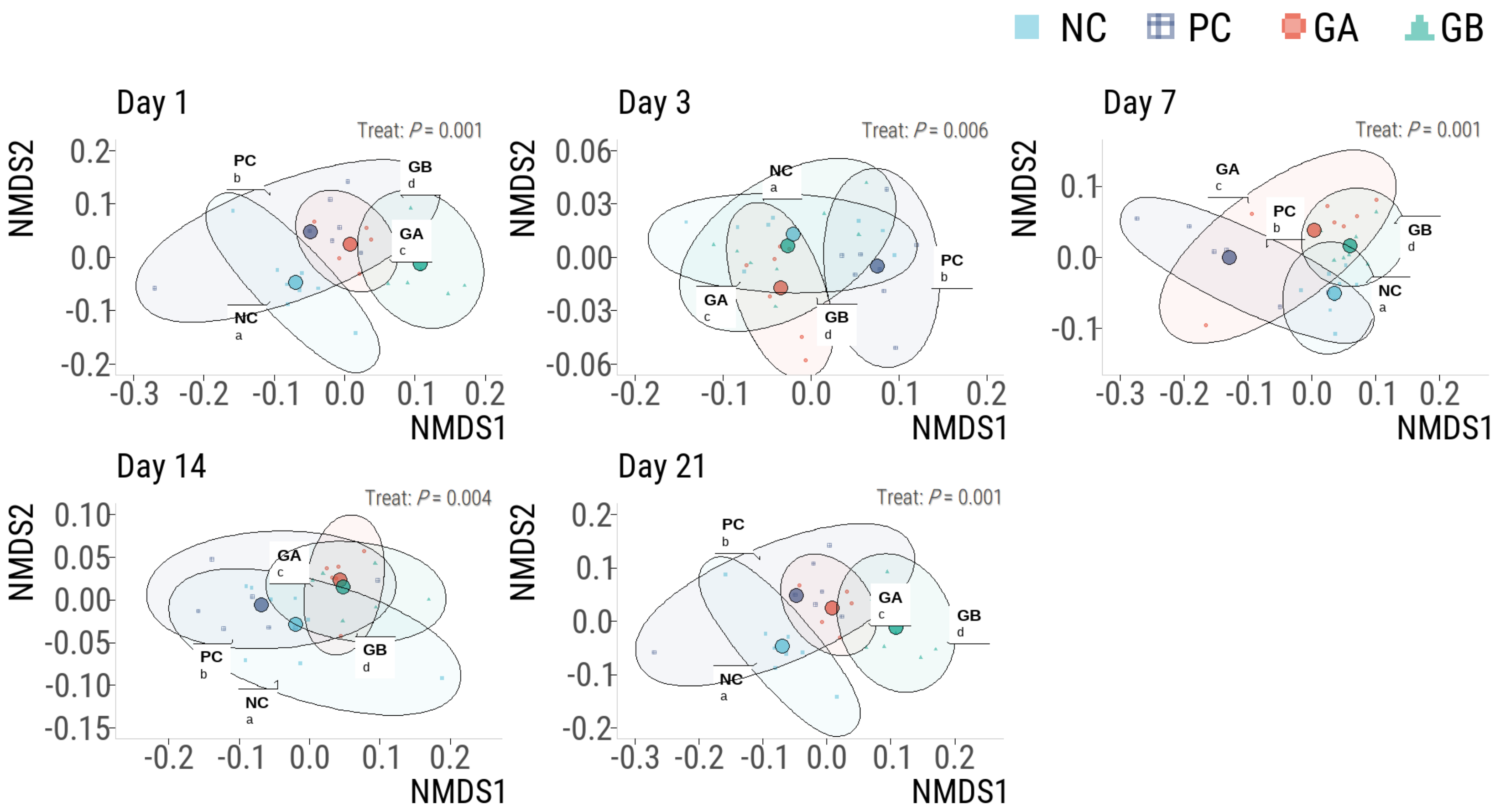

Fig S1 Effect of postweaning enterotoxigenic *E. coli* (ETEC) F18 challenge and supplementation with plant combinations on β-diversity measures of the fecal microbiota. Non-metric multidimensional scaling (NMDS) plot weighted UniFrac dissimilarity distances. Solid dots in ordination are mean centroids. [abcd] Groups that do not share a common superscript differ ($P < 0.05$), Benjamini-Hochberg adjustment. NC: non-challenge, standard diet (n=8); PC: challenged, standard diet (0-7d, n=8; 7-21d, n=7); GA: challenged, Garlic + Apple pomace (3%+3%; 0-7d, n=8; 7-21d, n=6); GB: challenged, Garlic + Blackcurrant (3%+3%; n=8).

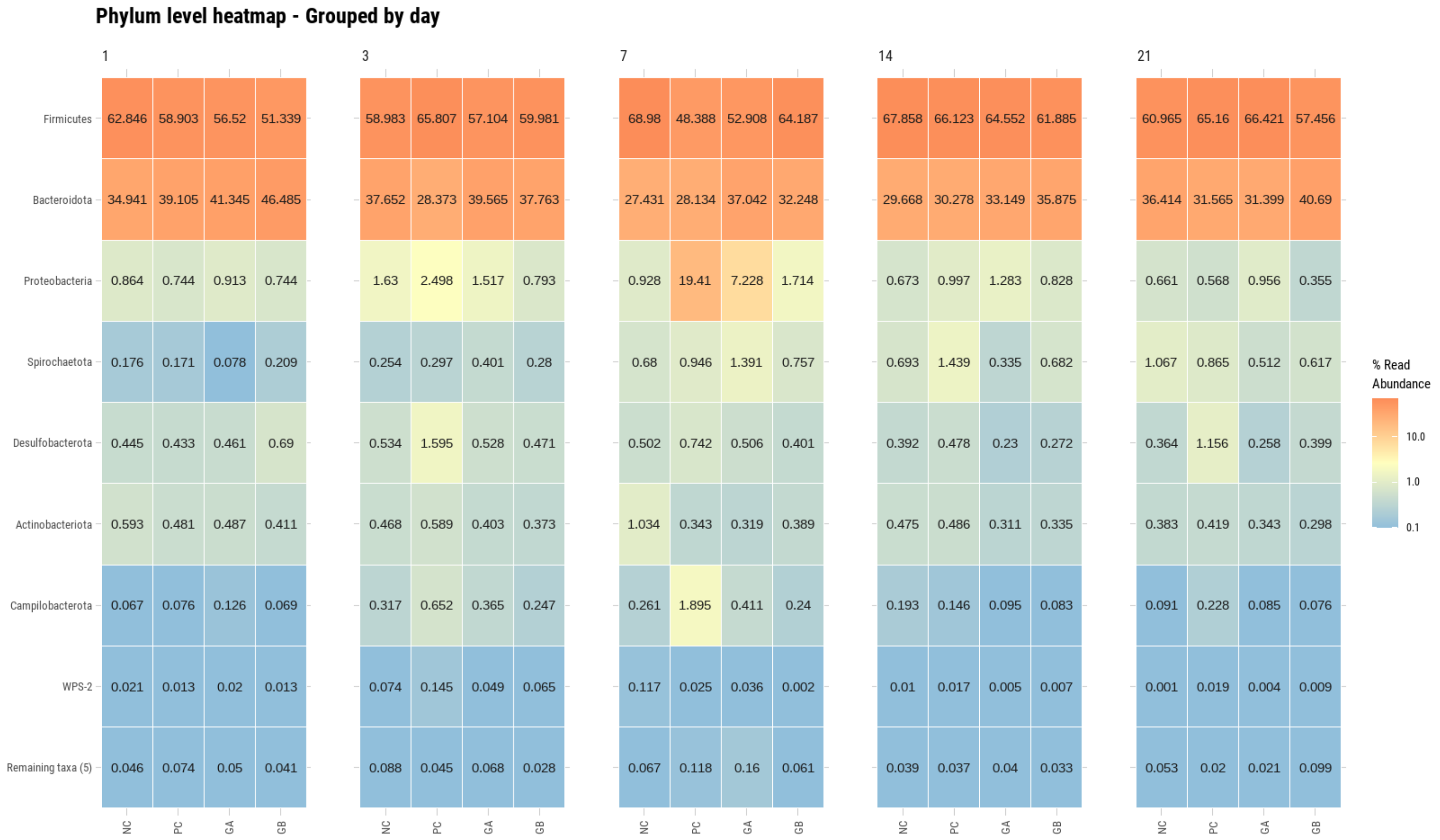

Fig S2 Relative abundance of bacterial genera within the feces of pigs challenged with enterotoxigenic *E. coli* (ETEC) F18 after weaning and supplemented with plant combinations. The ETEC F18 was administered orally on days 1 and 2. Values indicate mean relative abundance in percentage of the 20 dominant genera (Y-axis) across treatments (X-axis) and days postweaning (panel). NC: non-challenge, standard diet (n=8); PC: challenged, standard diet (0-7d, n=8; 7-21d, n=7); GA: challenged, Garlic + Apple pomace (3%+3%; 0-7d, n=8; 7-21d, n=6); GB: challenged, Garlic + Blackcurrant (3%+3%; n=8).

A

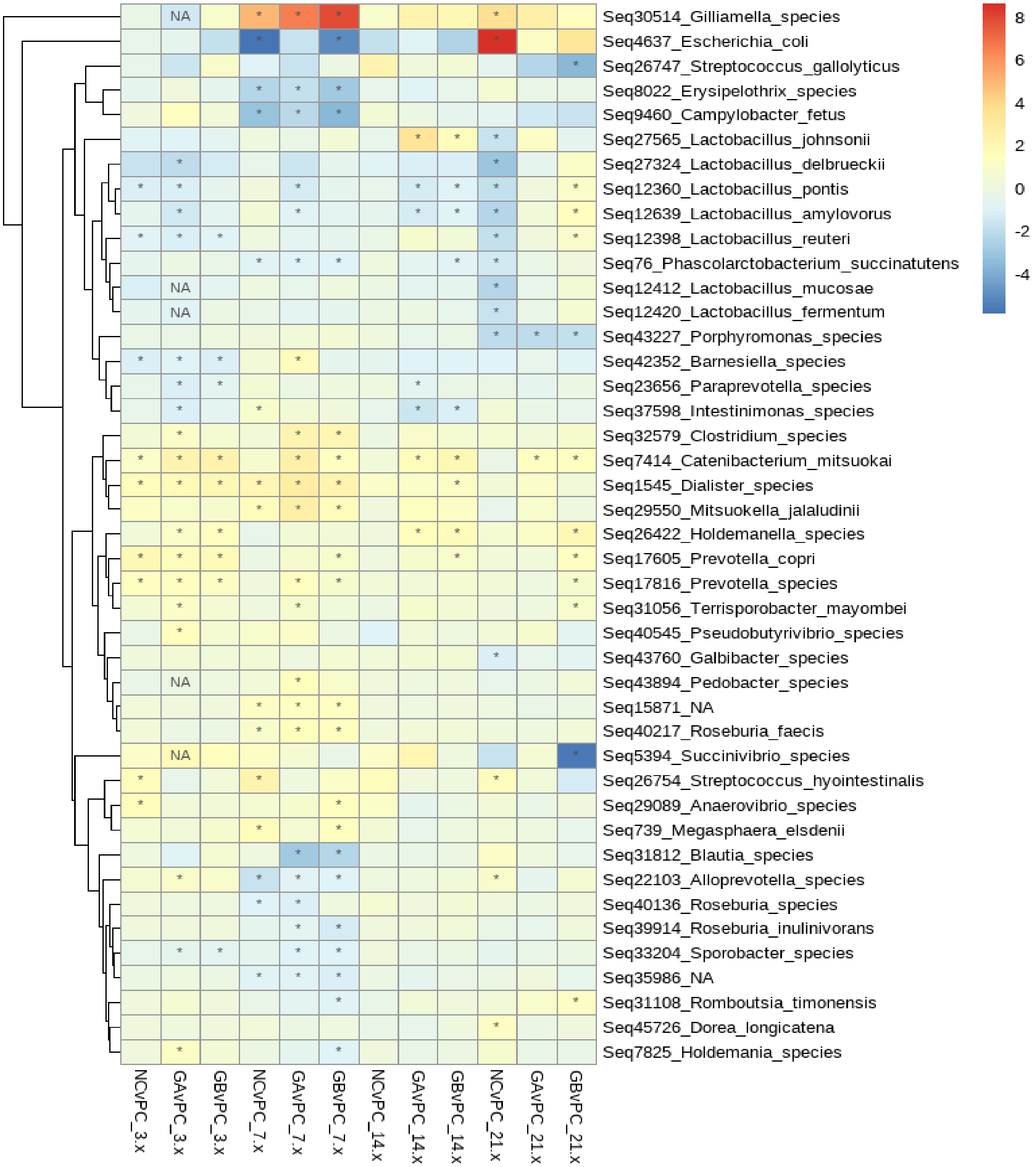

Fig S3 Differential abundance of bacterial genera within the feces of pigs challenged with enterotoxigenic E. coli (ETEC) F18 after weaning and supplemented with plant combinations. The ETEC F18 was administered orally on days 1 and 2. A: log2 fold-change difference from PC. (*): adj-P < 0.05 pairwise comparison against PC within day. NA: P value no estimable. B: DESeq2 normalized counts of differentially abundant genera. Only differentially abundant taxa (log2FC > 2; P < 0.05) are represented in the figures. NC: non-challenge, standard diet (n=8); PC: challenged, standard diet (0-7d, n=8; 7-21d, n=7); GA: challenged, Garlic + Apple pomace (3%+3%; 0-7d, n=8; 7-21d, n=6); GB: challenged, Garlic + Blackcurrant (3%+3%; n=8).

B

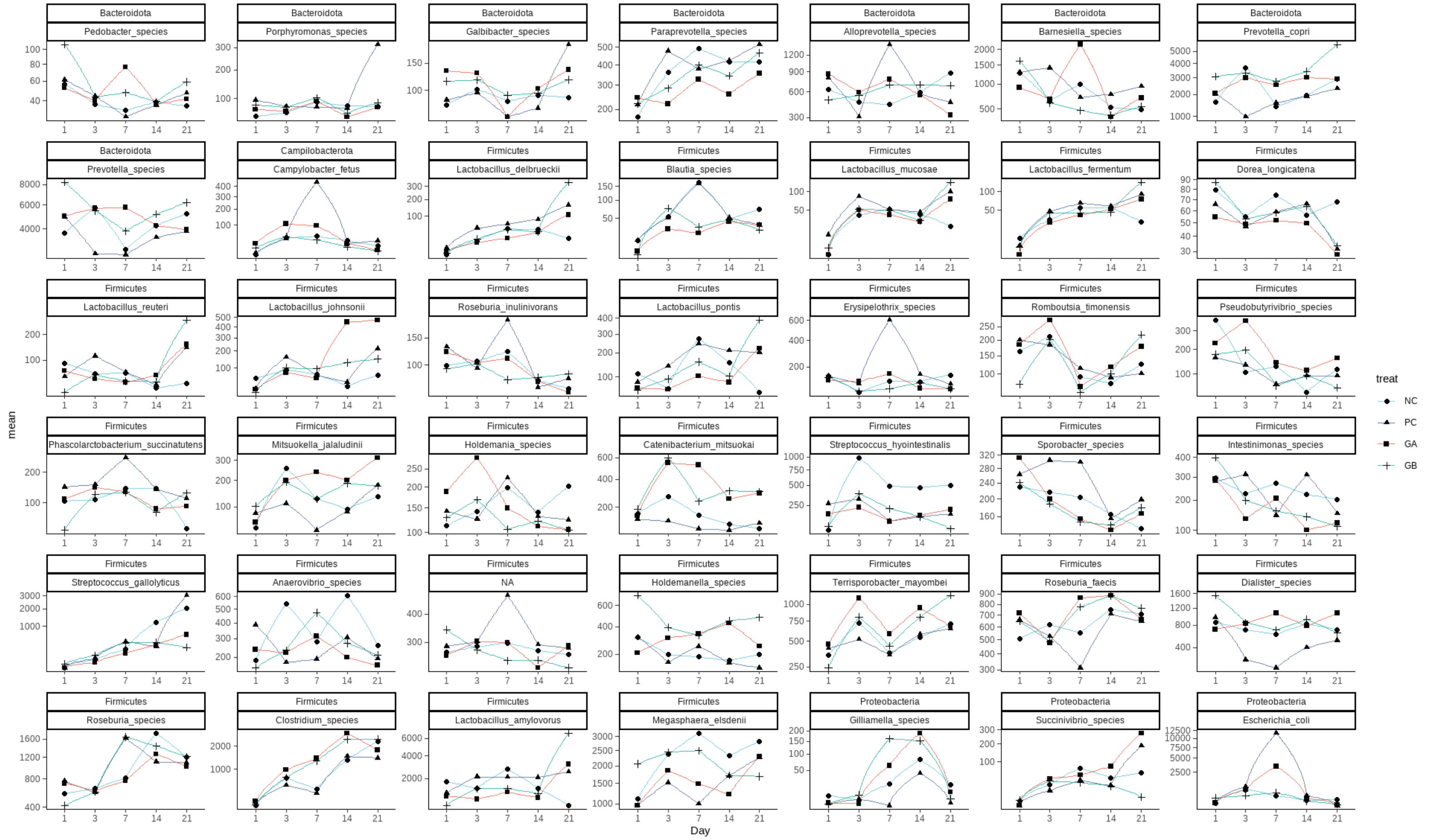

Fig S3 Differential abundance of bacterial genera within the feces of pigs challenged with enterotoxigenic E. coli (ETEC) F18 after weaning and supplemented with plant combinations. The ETEC F18 was administered orally on days 1 and 2. A: log2 fold-change difference from PC. (*): adj-P < 0.05 pairwise comparison against PC within day. NA: P value no estimable. B: DESeq2 normalized counts of differentially abundant genera. Only differentially abundant taxa (log2FC > 2; P < 0.05) are represented in the figures. NC: non-challenge, standard diet (n=8); PC: challenged, standard diet (0-7d, n=8; 7-21d, n=7); GA: challenged, Garlic + Apple pomace (3%+3%; 0-7d, n=8; 7-21d, n=6); GB: challenged, Garlic + Blackcurrant (3%+3%; n=8).
